# A Bioactive 3D-Bioprinted AuNC@DHLA/GelMA Hydrogel Containing Skin Cells Promotes Burn Wound Healing and Tissue Regeneration

**DOI:** 10.3390/polym18192331

**Published:** 2026-09-24

**Authors:** Kuang-Ling Ou, Chun-Ting Chen, Kai-Chieh Chou, Peng-Chi Jou, Juin-Hong Cherng, Yi-Wen Wang

**Affiliations:** 1 Division of Plastic and Reconstructive Surgery, Department of Surgery, Tri-Service General Hospital, National Defense Medical University, Taipei 114, Taiwan; kuanglio085@gmail.com; 2 Burn Center, Department of Surgery and Department of Critical Medicine, Tri-Service General Hospital, National Defense Medical University, Taipei 114, Taiwan; 3 Division of Gastroenterology and Hepatology, Department of Internal Medicine, Tri-Service General Hospital Penghu Branch, National Defense Medical University, Penghu County 880, Taiwan; gp0921543gp@yahoo.com.tw; 4 Division of Gastroenterology and Hepatology, Department of Internal Medicine, Tri-Service General Hospital, National Defense Medical University, Taipei 114, Taiwan; 5 Graduate Institute of Biology and Anatomy, College of Medicine, National Defense Medical University, Taipei 114, Taiwan; jeffrey8464@gmail.com (K.-C.C.); eric39326671@gmail.com (P.-C.J.); 6 Graduate Institute of Life Sciences, National Defense Medical University, Taipei 114, Taiwan; i72bbb@gmail.com

**Keywords:** burn injury, hydrogel, wound healing, nanoparticle, 3D bio-printing, tissue engineering, skin substitutes

## Abstract

Severe burn injuries are often associated with excessive and prolonged inflammation, resulting in delayed wound healing and poor tissue regeneration. Accordingly, the development of effective skin substitutes capable of modulating inflammation and promoting tissue repair remains a critical challenge in regenerative medicine. Gelatin methacryloyl (GelMA) has emerged as a promising biomaterial for tissue engineering applications. Moreover, dihydrolipoic acid-modified gold nanoclusters (AuNC@DHLA) have demonstrated anti-inflammatory and regenerative-promoting effects. In this study, we fabricated a bioactive tissue-engineered skin construct by incorporating AuNC@DHLA, fibroblasts, and keratinocytes into a 3D-bioprinted GelMA hydrogel scaffold. The therapeutic efficacy of the cell-laden AuNC@DHLA/GelMA scaffold was evaluated using a murine burn wound model. Furthermore, RNA sequencing was further conducted to explore the molecular mechanisms underlying wound repair, and the findings were subsequently validated by immunohistochemistry. The results demonstrated that the scaffold significantly attenuated local inflammatory responses and accelerated burn wound closure compared with control treatments. Transcriptomic analysis revealed the modulation of inflammation-related and tissue regeneration-associated pathways, which was further confirmed by immunohistochemical analyses. Collectively, these findings indicate that the AuNC@DHLA/GelMA bioengineered skin substitute promotes burn wound healing by regulating the inflammatory microenvironment and enhancing tissue regeneration. This strategy represents a promising therapeutic approach for the treatment of severe burn injuries.

## 1. Introduction

Large-area burn injury wounds disrupt the skin’s barrier function, increasing the risk of severe infection. When the damaged area is extensive, autologous skin grafting is often limited by the scarcity of healthy donor sites, necessitating the development of tissue-engineered skin substitutes to promote healing [1]. Three-dimensional (3D) bioprinting has emerged as an innovative technology in tissue engineering and regenerative medicine. By leveraging specific bioink properties—such as viscosity, shear-thinning behavior, and thermosensitivity—3D bioprinting allows the fabrication of complex architectures and functional scaffolds tailored to the specific shape, size, and depth of a patient’s wound. This technology serves as a powerful tool in biomedical research by precisely controlling cell distribution and fabricating extracellular matrix (ECM) microenvironments that mimic native tissues [2].

Anatomically, skin consists of the epidermis and the dermis. The epidermis is predominantly composed of keratinocytes, which proliferate and differentiate externally to form a waterproof stratum corneum. The dermis, consisting of fibroblasts and their secreted ECM, provides structural support. Beneath these layers, the hypodermis contains a rich microvascular network that extends into the dermis to supply essential oxygen and nutrients [3]. Utilizing bioinks with excellent biocompatibility, researchers can sequentially layer fibroblasts or stem cells to construct the dermis, followed by keratinocyte-encapsulating bioinks to replicate the epidermis. The resulting engineered skin not only covers the wound but also stimulates growth factor secretion, thereby accelerating granulation tissue formation and re-epithelialization [4]. Consequently, 3D bioprinting represents a promising approach for generating skin graft alternatives.

Wound healing progresses sequentially through four overlapping phases: hemostasis, inflammation, proliferation, and remodeling. Following clot formation, the wound enters the inflammatory phase, characterized by the infiltration of immune cells, such as neutrophils and macrophages. These cells clear bacteria and debris while releasing pro-inflammatory cytokines and growth factors that recruit endothelial cells and fibroblasts to the wound site [3,5]. During the proliferative phase, these cells rapidly expand to drive angiogenesis, restoring oxygen and nutrient supply to the wound bed [6,7]. As granulation tissue develops, keratinocytes migrate from the wound periphery and hair follicles across the newly formed tissue, proliferating and differentiating to achieve re-epithelialization [5]. However, excessive inflammation can disrupt the balance between pro- and anti-inflammatory regulation. This exacerbates the accumulation of neutrophils, leukocytes, and macrophages, leading to overproduction of inflammatory cytokines and reactive oxygen species (ROS). This oxidative stress hinders the migration and proliferation of fibroblasts and epithelial cells, delaying healing and potentially causing excessive scarring [8]. Therefore, skin scaffolds capable of modulating inflammation and stimulating cell proliferation are highly desirable for wound repair.

Gold nanoclusters (AuNCs) have demonstrated significant potential in modulating inflammation, reducing oxidative stress, and enhancing wound healing. Previous studies showed that AuNCs upregulate the target myogenic differentiation proteins MyoD, MyoG, and Tnnt-1, thereby promoting myotube formation, repairing skeletal muscle defects in rats, and significantly increasing capillary density [9]. AuNCs can be synthesized with dihydrolipoic acid (DHLA) [10], the reduced form of alpha-lipoic acid (ALA). Both DHLA and ALA exhibit potent antioxidant properties, including free radical scavenging and metal-chelating capabilities, which protect cells from oxidative damage and maintain cellular homeostasis [11,12,13]. Furthermore, the unique amphiphilic (lipid- and water-soluble) nature of lipoic acid enhances its bioavailability, facilitating efficient intracellular uptake [14]. Because burn injuries elicit more severe inflammatory responses than standard wounds, previous work incorporated DHLA-modified gold nanoclusters (AuNC@DHLA) into chitosan scaffolds in a rat burn model, observing suppressed TNF expression, modulated inflammation, and accelerated wound healing [15]. Our previous study also demonstrated that AuNC@DHLA promotes the proliferation of human dermal fibroblasts [16].

Despite their therapeutic efficacy, these ultra-small AuNCs must be integrated with a biomaterial matrix to ensure retention within the wound bed. Gelatin methacryloyl (GelMA), a modified gelatin polymer featuring excellent biocompatibility, is widely used in tissue engineering and biomaterial development [17]. To fabricate diverse scaffolds via 3D bioprinting, GelMA can encapsulate various elements, including nanomaterials and cells [18,19]. Therefore, this study aims to fabricate tissue-engineered skin by incorporating AuNC@DHLA, fibroblasts, and keratinocytes into a GelMA hydrogel and to investigate its impact on wound healing. We first determined the optimal therapeutic concentration of the AuNC@DHLA/GelMA hydrogel. The optimized cell-laden 3D-printed AuNC@DHLA/GelMA scaffolds were applied in a murine skin burn model, and RNA sequencing was employed to explore the underlying molecular mechanisms of wound healing, followed by validation via immunohistochemical staining. Collectively, these findings indicate that the AuNC@DHLA/GelMA bioengineered skin substitute promotes burn wound healing by regulating the inflammatory microenvironment and enhancing tissue regeneration. This strategy represents a promising therapeutic approach for the treatment of severe burn injuries and may have potential applications in both clinical wound management and military medicine.

## 2. Materials and Methods

### 2.1. Materials

AuNC@DHLA was provided by Dr. Cheng-An J Lin, who originally synthesized the AuNCs in the previous study [10]. Human fibroblasts (PCS-201-012) and HaCaT cells (#330493) were purchased from the American Type Culture Collection (ATCC, Manassas, VA, USA) and CLS Cell Line Service GmbH (Eppelheim, Germany), respectively. DAPI (4′,6-diamidino-2-phenylindole; #17513) was purchased from AAT Bioquest, Inc. (Pleasanton, CA, USA), and propidium iodide (PI; #40017) was purchased from Biotium, Inc. (Fremont, CA, USA). CellTracker Red CMTPX, CellTracker Blue CMAC, and Calcein AM were purchased from Thermo Fisher Scientific (Burlington, MA, USA). The following primary antibodies were purchased from GeneTex, Inc. (Irvine, CA, USA): rabbit anti-TNF-α (GTX110520), rabbit anti-α-SMA (GTX100034), rabbit anti- cleaved caspase-3 (GTX86952), goat anti-rabbit IgG (GTX213110-01), and mouse IgG (GTX213111-01). Mouse anti-IL-1β (#12242), rabbit anti-IL-10 (#12163), and rabbit anti-IL-6 (#12153) were purchased from Cell Signaling Technology, Inc. (Danvers, MA, USA). Rabbit anti-CD80 (ab254579) was purchased from Abcam (Cambridge, UK), and mouse anti-TGFβ1 (sc130348) was purchased from Santa Cruz Biotechnology, Inc. (Santa Cruz, CA, USA).

### 2.2. Synthesis of Gelatin Methacrylate (GelMA) and Determination of the Degree of Methacrylation (DoM)

GelMA was synthesized as previously described [20]. Briefly, 10 g of gelatin (G2500, Sigma-Aldrich, St. Louis, MO, USA) was dissolved in 100 mL of ultrapure water at 45–55 °C until a clear, homogeneous yellow solution was obtained. Methacrylic anhydride (6 mL; #276685, Sigma-Aldrich, Darmstadt, Germany) was then added dropwise along the inner wall of the beaker under continuous stirring, and the reaction was allowed to proceed for 1 h at 45–55 °C. The reaction was quenched by adding 100 mL of ultrapure water, followed by continuous stirring for an additional 3 h at the same temperature. The reaction mixture was transferred into dialysis tubing (molecular weight cutoff, 12–14 kDa) and dialyzed against ultrapure water for 5 days, with water replacement twice daily at 8-h intervals. The dialyzed GelMA solution was subsequently lyophilized and stored in a light-protected desiccator until use.

^1^*H* nuclear magnetic resonance (^1^*H* NMR) spectra of gelatin and GelMA were acquired using a 400 MHz JEOL NMR spectrometer (NM-70010S4L1) (JEOL, Peabody, MA, USA) equipped with a 5 mm broadband gradient autotunable probe. Samples were dissolved in D_2_O and analyzed at approximately 25 °C with 8 scans and a relaxation delay of 5 s. The DoM was estimated by comparing the integrated intensity of the methacrylate vinyl proton signals with that of the phenylalanine aromatic proton signals. The DoM was calculated using the phenylalanine-to-lysine molar fraction ratio of the parent gelatin as a reference according to the following equation:

$$DoM(\%)=\frac{I_{vinyl}/2}{I_{Phe}/5}\times \frac{f_{Phe}}{f_{Lys}}\times 100$$

### 2.3. Dynamic Light Scattering and Zeta Potential Analysis

The hydrodynamic size distribution and zeta potential of AuNC@DHLA were characterized using a Litesizer DLS 700 (Anton Paar, Styria, Austria). AuNC@DHLA were analyzed in pure water and 10% (*w*/*v*) GelMA, whereas GelMA-containing samples were prepared in phosphate-buffered saline (PBS). Measurements were performed at 50 °C to maintain GelMA in the sol state. Dynamic light scattering (DLS) measurements were performed at scattering angles of 15°, 90°, and 175°, with 60 processed runs per measurement. Particle-size distributions were evaluated on a volume-weighted basis. Zeta potential was determined by electrophoretic light scattering using an Omega cuvette (Anton Paar). A Henry factor of 1.5 was used for zeta-potential determination. Zeta-potential values are reported as mean ± standard deviation.

### 2.4. UV–Visible Spectrophotometry

The optical characteristics of AuNC@DHLA, GelMA, and AuNC@DHLA-loaded GelMA were evaluated using a CLARIOstar microplate reader (BMG LABTECH, Ortenberg, Germany). Absorbance spectra were recorded over the wavelength range of 300 to 730 nm.

### 2.5. Biocompatibility of AuNC@DHLA/GelMA Hydrogels

GelMA and Irgacure2959 were dissolved in ultrapure water to prepare a stock solution containing 20% (*w*/*v*) GelMA and 1% (*w*/*v*) Irgacure2959. This solution was then mixed at a 1:1 volume ratio with fresh culture medium containing cells and AuNC@DHLA to yield a final formulation of 10% (*w*/*v*) GelMA with AuNC@DHLA concentrations of 0, 10, 25, 50, or 100 nM. The cell density in each formulation was 1 × 10^6^ cells/mL for fibroblasts or HaCaT cells. A total of 200 μ L of each precursor solution was dispensed into each well of a 24-well plate and photocrosslinked under 365 nm UV irradiation at 10 mW/cm^2^ for 3 min, followed by culture in fresh medium. Cell viability was assessed on days 1 and 7. Cell nuclei were stained with DAPI for 12 h prior to imaging. PI was subsequently added to the culture medium, and samples were incubated for 15 min before imaging to identify dead cells. Cell viability was quantified from fluorescence images according to the following equation: Cell Viability (%) = [(number of DAPI^+^ cells − number of PI^+^ cells)/number of DAPI^+^ cells]×100%, where DAPI^+^ cells represent the total cell nuclei and PI^+^ cells represent dead or membrane-compromised cells.

### 2.6. Rheological Characterization of GelMA and AuNC@DHLA/GelMA Hydrogels

The viscosity of the GelMA precursor solutions was measured using a viscometer (DVNext, AMETEK Brookfield, Middleborough, MA, USA) to evaluate their shear-thinning behavior. Rheological measurements were performed using a TA ARES-G2-UV rheometer (TA Instruments, New Castle, DE, USA) with a 20 mm parallel-plate geometry at 25 °C and a gap distance of 1000 µm. Storage modulus (G′) and loss modulus (G″) were recorded throughout the measurements. For time sweep analysis, uncrosslinked precursor solutions were measured at a constant frequency of 1.0 Hz and strain of 1.0%, with a sampling rate of 1.0 point/s. UV irradiation was applied at 10 mW/cm^2^ after a 30 s delay, followed by 630 s of exposure. For frequency sweep analysis, photocrosslinked hydrogels were measured over an angular frequency range of 0.1–100 rad/s under the same temperature and geometry conditions. Each group was tested in triplicate (*n* = 3), and data are presented as mean ± standard deviation (SD).

### 2.7. 3D Bioprinting of GelMA and AuNC@DHLA/GelMA Scaffolds

Three-dimensional bioprinting of 10% (*w*/*v*) GelMA containing 0.5% (*w*/*v*) Irgacure2959, with or without 50 nM AuNC@DHLA, was performed using a Bio X bioprinter (Cellink, Gothenburg, Sweden). Printing parameters were selected based on preliminary trials to achieve continuous filament extrusion, reproducible scaffold geometry, and structural stability. The syringe barrel temperature was maintained at approximately 19 °C, and the printing platform was set at 13 °C. Cylindrical scaffolds (10 mm in diameter and 1 mm in height) were fabricated using a 25-gauge nozzle with a 0/90° grid pattern and four printed layers. To maintain continuous filament deposition according to the rheological behavior of the bioink, the printing speed and extrusion pressure were controlled within narrow ranges of 1.9–2 mm/s and 90–100 kPa, respectively. The estimated filament diameter was approximately 200 μm. After printing, the constructs were exposed to 365 nm UV light at an irradiance of 10 mW/cm^2^ for 3 min to induce photocrosslinking and stabilize the scaffold architecture (Scheme 1).

### 2.8. Tensile Properties of 3D-Printing GelMA and AuNC@DHLA/GelMA Scaffolds

The tensile properties of the 3D-printed scaffolds were evaluated using a micro tensile testing machine (FALCO, Szombathely, Hungary, FAL-548M1-M). Grid-like dumbbell-shaped scaffolds with 40 mm in length, 10 mm in width, and 1.6 mm in thickness were subjected to uniaxial tensile loading at a constant crosshead speed of 15 mm/min until failure. Force and extension were continuously recorded to generate force–extension curves. The maximum force and elongation at break were determined.

### 2.9. Pore Size Distribution and Porosity Analysis

Scaffold microstructure was characterized by scanning electron microscopy (SEM) using an Apreo 2 SEM system (Thermo Fisher Scientific). Representative SEM images were acquired at the same magnification for all groups, and pore morphology was quantitatively analyzed using ImageJ software (Version 1.54p, National Institutes of Health, Bethesda, MD, USA). Objects intersecting the image border were excluded from analysis. Scaffold porosity was determined using the liquid displacement method. Briefly, dry scaffolds were first weighed to obtain the dry weight (W_d)_ and then immersed in ethanol. After saturation, the scaffolds were removed, gently blotted to eliminate excess ethanol on the surface, and immediately weighed to obtain the saturated weight (W_s_). Porosity was calculated as follows:

$$Porosity\;(\%)=\frac{W_{s}-W_{d}}{\rho_{ethanol}\times V}\times 100$$

where *ρ*_ethanol_ is the density of ethanol, and *V* is the scaffold volume.

### 2.10. Swelling and Degradation Profiles of 3D-Printing GelMA and AuNC@DHLA/GelMA Scaffolds

The equilibrium swelling ratio (ESR) of GelMA and AuNC@DHLA/GelMA hydrogels was determined in phosphate-buffered saline (PBS, Thermo Fisher Scientific). After photocrosslinking, the hydrogels were freeze-dried to obtain the initial dry weight (*W_d_*). Each sample was then immersed in PBS at $3{7\;}^{\circ }C$ . At predetermined time points, the samples were removed from PBS, and excess surface liquid was gently blotted off using filter paper before weighing to obtain the swollen weight (*W_t_*). This procedure was repeated until no further significant change in sample weight was observed, and the final constant weight was defined as the equilibrium swollen weight (*W_s_*). The ESR was calculated using the following equation:

$$ESR\;(\%)=\frac{W_{s}-W_{d}}{W_{d}}\times 100$$

The apparent degradation profiles of GelMA and AuNC@DHLA/GelMA hydrogels were evaluated in PBS containing 1 U/mL type I collagenase (Thermo Fisher Scientific) at $3{7\;}^{\circ }C$ . After photocrosslinking, the initial wet weight of each hydrogel was recorded as *W*_0_. The samples were then immersed in the enzyme solution and collected at predetermined time points. Excess surface liquid was carefully blotted off with filter paper, and the wet weight was measured as *W_t_*. The apparent remaining mass was calculated as follows:

$$Apparentremainingmass\;(\%)=\frac{W_{t}}{W_{0}}\times 100$$

### 2.11. Fabrication of Cell-Laden GAC Scaffolds

To fabricate the cell-laden GAC scaffolds, 10% (*w*/*v*) GelMA solution containing fibroblasts (1 × 10^6^ cells/mL), with or without 50 nM AuNC@DHLA, was prepared as the bioink for the dermal layer and printed using a Bio X bioprinter under the same printing conditions described above. Following the photo-crosslinking, a 10% (*w*/*v*) GelMA hydrogel containing pre-labeled HaCaT cells (1 × 10^6^ cells/mL), with or without 50 nM AuNC@DHLA, was manually distributed over the upper surface of the crosslinked scaffold to mimic the epidermal layer, followed by an additional 30 s of UV exposure. Gross morphology and cell distribution within the printing filaments were observed by brightfield microscopy (Zess, Jena, Germany). Post-fabrication cell viability after the printing/crosslinking process was further assessed on day 3 using a Live/Dead assay. Briefly, cell-laden scaffolds were stained with Calcein AM for 2 h to label live cells and PI for 15 min to label damaged cells. Images were acquired using a THUNDER Imager (Leica, Wetzlar, Germany). To verify structural compartmentalization, human dermal fibroblasts and HaCaT keratinocytes were pre-labeled with CellTracker Red CMTPX and CellTracker Blue CMAC, respectively, before mixing with GelMA. On the day of imaging, live cells within the constructs were labeled with Calcein AM (green). The stratified, cross-sectional architecture and post-printing cell viability were comprehensively characterized using a THUNDER Imager equipped with computational clearing/digital deconvolution technology.

### 2.12. Murine Model for Burn Injuries

Six-week-old nude mice (BALB/cAnN.Cg-Foxn1nu/CrlNarl) were purchased from BioLASCO Taiwan Co., Ltd. (Yilan, Taiwan). All animal procedures were reviewed and approved by the National Defense Medical Center Institutional Animal Care and Use Committee (IACUC-22–257). Following anesthesia via intraperitoneal injection of Zoletil 50 (20 mg/kg; Virbac, Carros, France) in combination with xylazine (10 mg/kg) and antiseptic preparation of the dorsal skin, a cylindrical copper rod (0.9 cm diameter) preheated to 100 °C was applied to the exposed dorsal skin for 5 s to induce a second-degree thermal injury (day 0), according to a previously reported method [21]. The burn area was immediately covered with normal gauze and Tegaderm transparent film dressing (3 M Health Care, St. Paul, MN, USA) to prevent further self-inflicted damage. Mice were then housed individually under controlled environmental conditions for subsequent experimental analyses.

To histologically verify the severity of the induced burn injury, skin tissues were collected from representative animals on day 1 after burn induction and subjected to hematoxylin and eosin (H&E) staining.

Necrotic tissue was surgically debrided on day 1. The wounds were subsequently treated with gauze (lesion control, LC), a GelMA scaffold (GelMA group), an AuNC@DHLA/GelMA scaffold (GA group), a cell-laden GelMA scaffold without AuNC@DHLA (GC group), or a cell-laden AuNC@DHLA/GelMA scaffold (GAC group). Each group included five to six mice. For postoperative infection prophylaxis and analgesia, cefazolin (25 mg/kg; China Chemical & Pharmaceutical, Taipei, Taiwan) and ketoprofen (2.5 mg/kg; Astar, Hsinchu, Taiwan) were administered once daily by intraperitoneal injection until wound closure.

### 2.13. Measurement of Wound Closure

Wound quantification was performed as previously described [22]. Burn wounds were gently irrigated with sterile saline and photographed at predetermined time points (days 0, 1, 3, 7, 14, 21, 28, and 35). Wound areas were quantified using ImageJ software. Wound closure was assessed independently from digital images by two blinded evaluators and expressed as a percentage of the initial wound area. The wound closure rate was calculated based on the remaining open wound area using a standard formula.

### 2.14. Histological Analysis

Cutaneous wound tissues were collected on day 35 and preserved in 4% paraformaldehyde. After fixation, wound tissues were embedded in paraffin. Paraffin-embedded tissue sections (5 μm) were subjected to hematoxylin and eosin (H&E) staining and Masson’s trichrome staining using commercial kits (Merck Millipore, Darmstadt, Germany; ScyTek Laboratories, Logan, UT, USA) according to the manufacturers’ protocols. Stained sections were digitized using an automated whole-slide scanner (Axio Scan Z1, Zeiss, Oberkochen, Germany) for histological evaluation and image analysis.

### 2.15. RNA Extraction, RNA Sequencing, and Quantitative Real-Time PCR (qPCR)

Total RNA was extracted from tissue samples collected on day 14 using a TRIzol™ Reagent (15596018, Thermo Fisher Scientific), following the manufacturer’s instructions. A total of 2 μg RNA from each sample was utilized for RNA-seq, following the manufacturer’s protocol. Qualified RNA samples were then submitted to a commercial sequencing facility for library preparation and RNA sequencing. Differentially expressed genes (DEGs) were identified using a threshold of |log2 fold change| > 1 and a false discovery rate (FDR) < 0.05. The FDR-adjusted q-value represents the expected proportion of false-positive results among the genes identified as statistically significant. DEGs meeting these criteria were subsequently subjected to Ingenuity Pathway Analysis (IPA; Qiagen, Germantown, MD, USA). Enriched pathways with a *p*-value < 0.05 were considered statistically significant. For qPCR analysis, 2 μg of total RNA was reverse-transcribed into cDNA using the Transcriptor First Strand cDNA Synthesis Kit (04897030001, Roche, IN, USA). The resulting cDNA was amplified by PCR with gene-specific primers, and Actb was used as the internal reference for normalization. qPCR was performed using cDNA templates, specific primers, and Power SYBR Green PCR Mix (Applied Biosystems) on a CFX96 Real-Time System (BIO-RAD, Hercules, CA, USA). Primer sequences are listed in Table 1.

### 2.16. Immunohistochemical Evaluation

Wound tissues collected on day 14 were paraffin-embedded and sectioned at 5 μm. Sections were deparaffinized in xylene and rehydrated through a graded ethanol series. To reduce nonspecific binding, sections were blocked with 1% normal goat serum in PBS for 1 h and incubated with primary antibodies overnight at 4 °C. Following PBS washes, sections were incubated with biotinylated secondary antibodies for 1 h, and signal amplification was achieved using a VECTASTAIN ABC kit (Vector Laboratories, Burlingame, CA, USA). Chromogenic detection was performed using 3,3′-diaminobenzidine (DAB) in the presence of hydrogen peroxide, followed by hematoxylin counterstaining. The stained slides were digitized using an automated whole-slide scanner (Axio Scan Z1, Zeiss, Oberkochen, Germany), and immunohistochemical signals were subsequently analyzed.

### 2.17. Statistical Analysis

Data are presented as mean ± SD. Statistical analyses were performed using one-way analysis of variance (ANOVA) followed by Tukey’s post hoc test for multiple comparisons. Differences were considered statistically significant at *p* < 0.05. All statistical analyses were conducted using GraphPad Prism 10 software (GraphPad Software, La Jolla, CA, USA).

## 3. Results

### 3.1. DOM of GelMA Hydrogel

GelMA formation was confirmed by ^1^*H* NMR spectroscopy (Figure 1A). Characteristic methacrylate vinyl proton signals were observed at 5.3–5.7 ppm in GelMA but were negligible in unmodified gelatin. A weak resonance around 6.0 ppm was also observed in GelMA and was attributed to methacryloyl-related unsaturated protons or trace residual low-molecular-weight methacrylate species. Using the aromatic proton signals at 7.15–7.45 ppm as an internal reference, the total vinyl integral was 3.78, corresponding to a relative methacrylation index of 1.89. Based on the reported amino acid composition of porcine skin Type A gelatin, the estimated degree of methacrylation was 85–100%, indicating successful synthesis of highly methacrylated GelMA.

### 3.2. Physicochemical Characterization of AuNC@DHLA and AuNC@DHLA-loaded GelMA

Transmission electron microscopy (TEM) showed that the AuNC@DHLA were uniformly dispersed and exhibited ultrasmall particle sizes (Figure 1B). DLS analysis at scattering angles of 15°, 90°, and 175° consistently demonstrated a predominant volume-weighted AuNC@DHLA population below 10 nm (Figure 1C). The apparent distribution maximum shifted from approximately 3 nm at 15° to 4–6 nm at 90° and 6–7 nm at 175°. Minor populations at larger particle sizes were also detected. These findings indicate that the AuNC@DHLA predominantly existed as nanoscale species in aqueous suspension, with only a minor contribution from larger scattering populations.

AuNC@DHLA exhibited a near-neutral zeta potential of −0.43 ± 0.94 mV in water (Figure 1D). The zeta potentials of 10% GelMA alone and AuNC@DHLA -loaded GelMA were 3.3 ± 1.3 and 4.0 ± 2.2 mV, respectively, indicating that incorporation of AuNC@DHLA did not markedly alter the mean zeta potential of the GelMA formulation (Figure 1E). The corresponding zeta-potential distribution peaks were 2.4, −7.6, and 6.2 mV for AuNC@DHLA, GelMA, and AuNC@DHLA-loaded GelMA, respectively, suggesting a shift in the distribution profile after AuNC@DHLA incorporation.

UV–visible spectroscopy further demonstrated increased broad optical absorption after incorporation of AuNC@DHLA into GelMA (Figure 1F), without the emergence of a distinct LSPR band at approximately 520 nm. Together with the DLS findings, the absence of a prominent 520-nm plasmonic band supports the presence of predominantly nanoscale AuNC species without evidence of substantial formation of larger plasmonic gold nanoparticles.

### 3.3. Optimization of AuNC@DHLA Concentration for Cell-Laden GelMA Hydrogels

To evaluate the biocompatibility of AuNC@DHLA within cell-laden GelMA matrix, the effects of various AuNC@DHLA concentrations on human dermal fibroblasts and HaCaT keratinocytes were examined. The 50 nM AuNC@DHLA group maintained significantly higher cell viability in both fibroblasts and HaCaT cells on days 1 and 7 than the other concentration groups (Figure 2 and Figure 3). Based on these findings, 50 nM AuNC@DHLA was selected for subsequent experiments.

### 3.4. Printability and Rheological Characterization of AuNC@DHLA/GelMA Hydrogel

Viscosity analysis demonstrated that both 10% GelMA and AuNC@DHLA mixed 10% GelMA precursor solutions exhibited distinct shear-thinning behavior, as evidenced by decreasing viscosity with increasing shear rate (Figure 4A). This non-Newtonian property supported favorable extrudability and enabled precise fabrication of 3D-printed grid scaffolds with high structural fidelity.

Time sweep analysis showed that both 10% GelMA and 10% GelMA + AuNC@DHLA underwent rapid photocrosslinking under UV irradiation, as evidenced by a marked increase in G′, maintenance of G′ > G″, and an increase in complex viscosity (Figure 4B,C). These changes indicate a transition from a liquid-like precursor to a highly elastic and structurally developed hydrogel network. The final G′ value of 10% GelMA was higher than that of 10% GelMA + AuNC@DHLA, indicating greater stiffness after gelation. In frequency sweep measurements, both hydrogels exhibited predominantly elastic behavior with G′ > G″ across the tested range, whereas 10% GelMA consistently showed higher moduli than 10% GelMA + AuNC@DHLA (Figure 4D).

### 3.5. Tensile Properties of AuNC@DHLA/GelMA 3D-Printed Scaffolds

Representative force–extension curves of the GelMA and AuNC@DHLA/GelMA scaffolds are shown in Figure 4E. Both scaffolds exhibited an initial increase in tensile force with increasing extension, followed by a decrease in force associated with structural failure. The GelMA scaffold exhibited a higher mean maximum force than the AuNC@DHLA/GelMA scaffold (0.106 ± 0.0169 vs. 0.0767 ± 0.0267 N, respectively) (Figure 4F), although the difference was not statistically significant. Conversely, the AuNC@DHLA/GelMA scaffold exhibited greater extension at maximum force (69.18 ± 28.72%) than the GelMA scaffold (47.74 ± 7.75%) (Figure 4G), but this difference also did not reach statistical significance.

### 3.6. Pore Size Distribution and Porosity of 3D-Printed GelMA and AuNC@DHLA/GelMA Scaffolds

SEM-based morphometric analysis demonstrated distinct differences in pore architecture between GelMA and AuNC@DHLA/GelMA scaffolds (Figure 5A,B). After excluding particles with an area smaller than 100 μm^2^, 81 and 79 pore profiles were analyzed for GelMA and AuNC@DHLA/GelMA, respectively. GelMA exhibited a mean pore area of 582.8 ± 555.3 μm^2^ and an equivalent circular diameter (ECD) of 24.8 ± 11.3 μm, whereas AuNC@DHLA/GelMA showed a mean pore area of 838.3 ± 1262.7 μm^2^ and an ECD of 28.1 ± 16.8 μm (Figure 5C,D). Although the difference in mean ECD was modest, AuNC@DHLA/GelMA displayed a markedly broader ECD distribution (11.6–110.9 μm) than GelMA (11.4–59.9 μm), suggesting substantially greater structural heterogeneity.

This interpretation was further supported by pore-shape descriptors. Comapred with GelMA, AuNC@DHLA/GelMA showed lower circularity (0.31 ± 0.14 vs. 0.61 ± 0.12), together with a higher aspect ratio (2.20 ± 1.00 vs. 1.63 ± 0.48) and larger Feret diameter (47.6 ± 38.32 μm vs. 33.1 ± 14.6 μm, *p* = 0.0021) (Figure 5E). These findings indicate that AuNC@DHLA incorporation altered the apparent pore geometry toward more elongated, irregular, and heterogeneous pore profiles in SEM cross-sections.

Porosity analysis by the liquid displacement method further showed that AuNC@DHLA incorporation significantly increased scaffold porosity. GelMA scaffolds showed a porosity of 62.7 ± 9.8%, while AuNC@DHLA/GelMAscaffolds exhibited a significantly higher porosity of 88.4 ± 6.4% (*p* = 0.007), indicating the formation of a more porous internal architecture (Figure 5F).

### 3.7. Water Uptake Capacity and Enzymatic Degradation Behavior of 3D-Printed GelMA and AuNC@DHLA/GelMA Scaffolds

The swelling ratio of GelMA and AuNC@DHLA/GelMA hydrogels in PBS increased rapidly during the initial incubation period and reached a maximum at 60 min (Figure 5G). At this time point, the equilibrium swelling ratio of AuNC@DHLA/GelMA hydrogels (643.95 ± 36.5%) was higher than that of GelMA hydrogels (474.1 ± 33.4%). Thereafter, both groups showed a slight decrease and gradually approached equilibrium. Two-way ANOVA revealed a significant effect of hydrogel composition on swelling behavior (*p* < 0.0001), indicating that AuNC@DHLA incorporation markedly enhanced the water uptake capacity of the GelMA hydrogel.

The apparent degradation profiles of GelMA and AuNC@DHLA/GelMA hydrogels were evaluated in PBS containing 1 U/mL type I collagenase. Both groups showed a time-dependent decrease in remaining mass, indicating progressive enzymatic degradation (Figure 5H). Compared with GelMA, the AuNC@DHLA/GelMA hydrogels exhibited a faster reduction in remaining mass throughout the incubation period and reached near-complete degradation at an earlier time point. Two-way ANOVA revealed a significant difference between the two groups (*p* < 0.0001), suggesting that AuNC@DHLA incorporation accelerated the apparent enzymatic degradation behavior of the GelMA hydrogel.

### 3.8. Cellular Distribution of AuNC@DHLA/GelMA Bioinks

3D bioprinting of 10% GelMA + AuNC@DHLA hydrogel yielded a well-defined 0/90° grid scaffold with grossly preserved macroscopic morphology (Figure 6A). Light microscopy showed that cell encapsulation did not compromise printing quality and that the cells were uniformly distributed throughout the hydrogel scaffold (Figure 6B). Live/Dead staining further confirmed that most encapsulated cells remained viable on day 3. (Figure 6C). To assess structural compartmentalization, cells were pre-labeled with fluorescent cell trackers before encapsulation. Three-dimensional reconstruction image demonstrated a well-organized stratified architecture in which keratinocytes (blue) and dermal fibroblasts (red) maintained their distinct layered distribution, while Calcein AM staining (green) confirmed post-printing cell viability (Figure 6D). These results indicate that incorporation of AuNC@DHLA preserved the favorable printability of GelMA and supported the construction of biomimetic, cell-viable skin substitutes.

### 3.9. 3D-Printed AuNC@DHLA/GelMA Scaffolds Accelerate Burn Wound Healing

To evaluate the in vivo therapeutic performance of the cell-laden AuNC@DHLA/GelMA scaffold (GAC group), the constructs were implanted into a murine burn wound model. Macroscopically, wounds treated with the GA, GC, and GAC scaffolds appeared smaller than those in the lesion control (LC) and GelMA groups on days 14, 21, and 28 (Figure 7A). Histological examination was performed to evaluate the extent and depth of thermal tissue damage and to confirm the characteristics of a deep second-degree burn injury (Figure 7B,C). Quantitative analysis further revealed that the GC and GAC groups achieved a wound-closure rate of 93.22% and 94.77%, respectively, on day 28, which were significantly higher than those in the LC (73.69%) and GelMA (65.84%) groups (Figure 7D).

### 3.10. Transcriptomic Profiling Revealed That GAC Promoted Cutaneous Repair by Activating Tissue Regeneration and Suppressing Inflammatory Pathways

To discover the potential biofunction underlying GAC-mediated wound repair, RNA sequencing was performed to profile the transcriptomic changes, followed by IPA to identify significantly altered canonical pathways (Figure 8A). Enrichment analysis revealed robust activation of pathways related to structural repair and functional tissue maturation, as indicated by positive z-scores.

Notably, the wound healing signaling pathway was significantly enriched and activated. Consistent with accelerated tissue remodeling, pathways governing extracellular matrix (ECM) organization were predominantly upregulated, including collagen biosynthesis and modifying enzymes, elastic fiber formation, collagen chain trimerization, and assembly of collagen fibrils and other multimeric structures. In parallel, pathways involved in cellular dynamics, such as activation of matrix metalloproteinases (MMPs) and myogenesis, were also activated, suggesting enhanced cellular migration and contractile myofibroblast differentiation.

Conversely, GAC treatment suppressed several hyperinflammatory pathways, as reflected by negative z-scores. Among these, the pyroptosis signaling pathway, a key regulator of inflammatory cell death, showed the strongest negative z-score among the downregulated pathways. This suppression was accompanied by inhibition of classical innate immune signaling pathways, including NOD-like receptor (NOD1/2) signaling and Toll-like receptor (TLR) signaling. In addition, pathways associated with oxidative stress and cytotoxicity, including production of nitric oxide and reactive oxygen species (ROS) in macrophages, TNF receptor-associated signaling, and the apoptotic execution phase, were also significantly downregulated.

### 3.11. Coordinated Regulation of Epithelial–Mesenchymal Transition and Inflammatory Response Pathways in GAC-Treated Wounds

To further dissect how the cell-laden 3D-bioprinted scaffold modulated the multicellular wound microenvironment, comparative gene set enrichment analysis (GSEA) was performed between the GAC group and the cell-free GelMA control group. The GAC group exhibited marked enrichment of the “hallmark epithelial mesenchymal transition” gene set (Figure 8B), with a strong normalized enrichment score (NES = 2.746) and high statistical significance (nominal *p* = 0.05; FDR q-value = 0.0). Leading-edge analysis further identified key regenerative and migratory drivers within the core enrichment subset (Table S1). Notably, interleukin-6 (Il6), pentraxin 3 (Ptx3), and the chemokines C-X-C motif chemokine ligand 5 (Cxcl5) and Cxcl12 ranked among the top positions in the gene list, indicating an activated cellular microenvironment primed for cell recruitment and migration. Furthermore, critical growth factors and matrix-remodeling enzymes, including matrix metalloproteinases (Mmp2, Mmp3, and Mmp14), tissue inhibitor of metalloproteinases 1 (Timp1), and transforming growth factor-beta 1 (Tgfb1), were prominently enriched. This pattern was accompanied by enrichment of structural ECM components and cell–matrix interaction molecules, such as vimentin (Vim), periostin (Postn), Sparc, and various collagen lineages (Col1a1, Col1a2, Col3a1, Col5a1, and Col5a2).

In parallel, the “hallmark inflammatory response” gene set was also significantly enriched in the GAC group (Figure 8C; NES = 1.870, nominal *p* = 0.0, FDR q-value = 0.0055, FWER *p*-value = 0.033). Leading-edge analysis identified key molecular drivers within the core-enrichment subset, suggesting coordinated regulation of inflammatory responses associated with tissue remodeling (Table S2). Notably, *Il6* and the neutrophil-recruiting chemokine *Cxcl5* ranked among the top genes in the gene list (Rank 0 and Rank 7, respectively; Rank Metric Scores = 32.460 and 8.528), suggesting their potential roles as key regulatory mediators. This enrichment was accompanied by increased representation of chemokines and leukocyte–endothelial adhesion molecules involved in directed cell recruitment, including *Sele* (*E-selectin*), *Ccl5*, and *Ccl7*, as well as *Csf3* (*granulocyte colony-stimulating factor*) and *Ptx3*. Collectively, these findings suggest the establishment of a pro-healing granulation tissue microenvironment.

To validate the GSEA findings, representative genes associated with epithelial–mesenchymal transition and inflammatory response pathways were quantified by qPCR. Key repair- and remodeling-associated markers, including IL6, Ccl7, Ptx3, and Tgfb1, exhibited the highest expression levels in the GAC group, whereas Csf3 was also elevated compared with the lesion control group. In contrast, the endothelial adhesion molecule Sele and the chemokines Cxcl5 and Ccl5 were prominently downregulated in GAC-treated tissues relative to the control. Together, these results indicate that the GAC formulation reshaped the wound microenvironment by promoting a selective cytokine and matrix-remodeling program while attenuating classical leukocyte-homing and proinflammatory mediators (Figure 8D).

### 3.12. Effects of the AuNC@DHLA/GelMA Scaffold and the Cell-Laden 3D-Printed AuNC@DHLA/GelMA Scaffold on Collagen Deposition and Dermal Remodeling

To verify the transcriptomic findings at the tissue level, histological analyses were performed to assess wound repair outcomes among the experimental groups. Hematoxylin and eosin (H&E) staining showed marked epidermal hyperplasia and excessive granulation tissue formation in the LC and GelMA groups. In contrast, the GA, GC, and GAC groups exhibited epidermal and granulation tissue thicknesses that were restored to levels comparable to those of adjacent normal tissue (Figure 9A). Masson’s trichrome staining further demonstrated that the intensity of blue-stained collagen fibers within the granulation tissue was greater in the GA and GAC groups than in the LC and GelMA groups (Figure 9B). To further validate these observations, the expression of fibroblast proliferation- and activation-associated markers, transforming growth factor-β (TGF-β) and alpha-smooth muscle actin (α-SMA) [23], was assessed by immunohistochemistry (IHC). IHC staining showed that both TGF-β and α-SMA were significantly upregulated in the GA and GAC groups relative to the LC and GelMA groups (Figure 9C).

### 3.13. Effects of AuNC@DHLA/GelMA Scaffolds and Cell-Laden 3D-Printed AuNC@DHLA/GelMA Scaffolds on Inflammatory Responses and Apoptosis

To investigate the effects of the AuNC@DHLA/GelMA scaffold and cell-laden 3D-printed AuNC@DHLA/GelMA scaffold treatments on post-burn inflammatory response, IHC staining was performed for CD80, a classic M1 macrophage biomarker [24]. CD80 expression was significantly decreased in both the GA and GAC groups compared with the other groups (Figure 10). We further quantified the expression of pro-inflammatory cytokines, including TNF-α, IL-1β, and IL-6, as well as the anti-inflammatory cytokine (IL-10) [24]. IL-1β and IL-10 expression levels were significantly reduced in the GA, GC, and GAC groups. In addition, the GC and GAC groups showed significantly lower TNF-α expression but higher IL-6 than the other groups (Figure 10). Finally, to evaluate the effects of the different treatments on apoptosis, cleaved caspase-3 expression was examined. Although cleaved caspase-3 levels tended to be lower in the GA and GAC groups than in the LC, GelMA, and GC groups, the differences did not reach statistical significance (Figure 10).

## 4. Discussion

Severe burn injury remains a major clinical challenge because of extensive tissue loss, persistent inflammation, and incomplete dermal regeneration. In this study, we developed a cell-laden 3D-bioprinted AuNC@DHLA/GelMA scaffold and demonstrated that this construct significantly improved burn wound healing. By integrating materials characterization, in vivo wound evaluation, transcriptomic profiling, gene set enrichment analysis, and histological validation, we found that this platform promoted skin repair through coordinated regulation of inflammatory signaling, epithelial–mesenchymal transition, and extracellular matrix remodeling.

A key finding of this study is that incorporation of AuNC@DHLA did not compromise the essential processing characteristics of GelMA as a printable hydrogel system. The synthesized GelMA exhibited a high degree of methacrylation, providing a stable photocrosslinkable polymer network suitable for extrusion-based bioprinting. After AuNC incorporation, the precursor retained nanoscale dispersion, shear-thinning behavior, and rapid photo-induced gelation, thereby supporting extrusion fidelity and shape retention during printing. Although AuNC@DHLA modestly reduced post-crosslinking stiffness, it significantly increased porosity, water uptake, and collagenase-sensitive degradation, suggesting altered network organization and enhanced matrix remodeling potential. SEM images qualitatively confirmed open porous scaffold structures; however, because SEM provides only two-dimensional information, pore interconnectivity could not be directly determined. Accordingly, these findings support the interpretation that AuNC incorporation generated a more permeable and dynamically remodelable matrix, which is advantageous for mass transport, cell accommodation, and scaffold turnover in skin tissue engineering. The favorable cytocompatibility of this material system further supports its suitability as a bioink platform.

Gold nanomaterials have attracted broad interest in regenerative medicine owing to their chemical stability, tunable surface properties, and generally favorable biocompatibility [25]. Previous studies have shown that gold-based nanostructures can influence cell adhesion, proliferation, angiogenic signaling, and oxidative stress responses [26,27]. However, these effects are strongly dose-dependent, and excessive nanoparticle exposure may impair cell viability [28]. In the present study, AuNC@DHLA at 50 nM provided the most favorable cytocompatibility in both fibroblasts and keratinocytes, indicating that this concentration achieved an appropriate balance between nanomaterial-associated bioactivity and cell tolerance. This optimization step is important because the translational utility of a nanocomposite bioink depends not only on printability, but also on its ability to preserve cell viability during and after encapsulation.

The improved wound healing efficacy observed in the GAC group likely reflects the synergistic interaction among the polymer scaffold, embedded skin cells, and AuNC-mediated modulation of the wound microenvironment. GelMA provides an ECM-mimetic matrix with suitable hydration, structural support, and biochemical permissiveness [29,30]. Encapsulated fibroblasts and keratinocytes may further enhance repair by delivering paracrine signals that regulate cell migration, matrix synthesis, and tissue regeneration [31]. Within this composite system, AuNCs may serve as functional nanoscale modifiers that reshape local physicochemical and biological conditions. Therefore, the therapeutic performance of the GAC scaffold is unlikely to arise from a single component, but rather from the coupled effects of polymer network design, cell delivery, and nanomaterial-enabled bioactivity.

The transcriptomic data provide important mechanistic support for this interpretation. Treatment with the cell-laden AuNC@DHLA/GelMA scaffold induced activation of wound healing and tissue remodeling pathways, including collagen biosynthesis, elastic fiber formation, collagen fibril assembly, myogenesis, and MMP-related pathways. These signaling programs are closely associated with dermal reconstruction, provisional matrix replacement, and maturation of granulation tissue [32,33]. The enrichment of myogenesis-related pathways is also consistent with fibroblast-to-myofibroblast transition, which contributes to wound contraction and matrix deposition during the proliferative stage of repair. At the same time, several inflammatory pathways were suppressed, including pyroptosis, Toll-like receptor signaling, and NOD1/2-related innate immune signaling. Because pyroptosis amplifies tissue injury through inflammasome-mediated inflammatory cell death [34,35], its inhibition may help prevent persistent inflammatory damage in the burn wound environment. Overall, these data suggest that the scaffold promoted a reparative molecular program while limiting excessive innate immune activation.

The GSEA results further indicate that the observed inflammatory signature should be interpreted as a regulated pro-healing response rather than uncontrolled inflammation. The GAC group showed enrichment of both the EMT and inflammatory response gene sets, with overlap in several key leading-edge genes. In particular, IL-6 and Cxcl5 emerged as shared nodes linking inflammatory activity with cell migration and remodeling [36,37]. Additional high-ranking genes, including Ptx3, Csf3, Tnfaip6, Sele, Ccl5, and Ccl7, suggest active control of immune cell recruitment, inflammatory resolution, and vascular-endothelial interactions [38,39,40]. Taken together, these findings indicate that the scaffold did not simply suppress inflammation globally, but rather redirected the wound response toward a controlled regenerative state that supports EMT-associated remodeling and dermal maturation.

Histological and immunohistochemical analyses were consistent with the transcriptomic findings. Compared with untreated wounds and GelMA alone, the GA and GAC groups exhibited more normalized epidermal architecture and less excessive granulation tissue. Increased collagen deposition in Masson’s trichrome staining, together with elevated TGF-β and α-SMA, indicates enhanced fibroblast activation, matrix synthesis, and myofibroblast-associated remodeling. These observations support the view that AuNC-containing scaffolds promote more effective dermal reconstruction at the tissue level.

The immunomodulatory effect of the scaffold is also noteworthy. The reduction in CD80-positive macrophages suggests attenuation of M1-like pro-inflammatory macrophage activation, which is consistent with the decreased expression of TNF-αand IL-1β. Interestingly, IL-6 expression was increased in the GAC group. Although IL-6 is commonly regarded as a pro-inflammatory cytokine, it also plays important roles in keratinocyte migration, fibroblast activation, angiogenesis, and regenerative signaling during wound healing [41]. In this context, elevated IL-6 may reflect a temporally appropriate regenerative response rather than unresolved inflammation. This interpretation is supported by the concomitant reduction in CD80 and classical inflammatory mediators, together with the overall enhancement of tissue repair.

From an integrated structure–property–function perspective, the present data support a multicomponent mechanism for the therapeutic activity of the GAC scaffold. The GelMA network provides a printable and photocrosslinkable polymer framework; AuNC incorporation modulates network architecture, degradation behavior, and inflammatory signaling; and embedded skin cells provide biologically active repair cues [26,30]. The convergence of these material and cellular functions likely underlies the coordinated activation of EMT-related remodeling, fibroblast-to-myofibroblast transition, ECM reconstruction, and wound closure observed in this study.

Several limitations should be acknowledged. First, although the transcriptomic analyses identified multiple pathways associated with regeneration and inflammation, causal validation of specific mechanisms, such as pyroptosis inhibition, EMT activation, and macrophage polarization, was not performed. Future studies using pathway inhibition, gene silencing, or cell-specific functional assays will be necessary to define these mechanisms more directly. Second, the present study focused primarily on wound closure, dermal remodeling, and inflammatory regulation, whereas vascularization and innervation within the regenerated tissue were not investigated. Because both are essential for recovery of full skin function, they should be included in future evaluations.

The present findings also support the translational potential of the AuNC@DHLA/GelMA system for burn wound treatment. AuNC@DHLA/GelMA-based bioinks can be produced through relatively standardized synthesis and photocrosslinking procedures, and the favorable rheological properties and printing fidelity observed here suggest compatibility with scalable manufacturing workflows. In practical terms, an acellular AuNC@DHLA/GelMA scaffold may offer advantages in shelf life, storage, transport, and regulatory approval, whereas cell-laden constructs may provide greater biological potency but require stricter control of cell sourcing, viability, sterility, and lot release criteria. Therefore, despite the encouraging in vitro and in vivo findings, further studies on storage stability, sterilization compatibility, long-term biosafety, and regulatory classification will be necessary before clinical translation.

## 5. Conclusions

In conclusion, this study demonstrates that AuNC@DHLA incorporation enables the development of a multifunctional GelMA-based bioink for 3D bioprinting of skin regenerative scaffolds. The resulting cell-laden construct combined favorable printability, cytocompatibility, and tunable physicochemical properties with enhanced burn wound healing efficacy in vivo. Mechanistically, the scaffold promoted repair through coordinated modulation of inflammatory responses, EMT-associated remodeling, and dermal matrix reconstruction. These findings highlight the value of integrating nanomaterial-enabled bioactivity, polymer network engineering, and cell-laden biofabrication in the design of next-generation skin substitutes. Further studies on vascularization, scar quality, large-animal validation, and scalable manufacturing will help advance the clinical translation of this platform.

## Figures and Tables

**Scheme 1:**
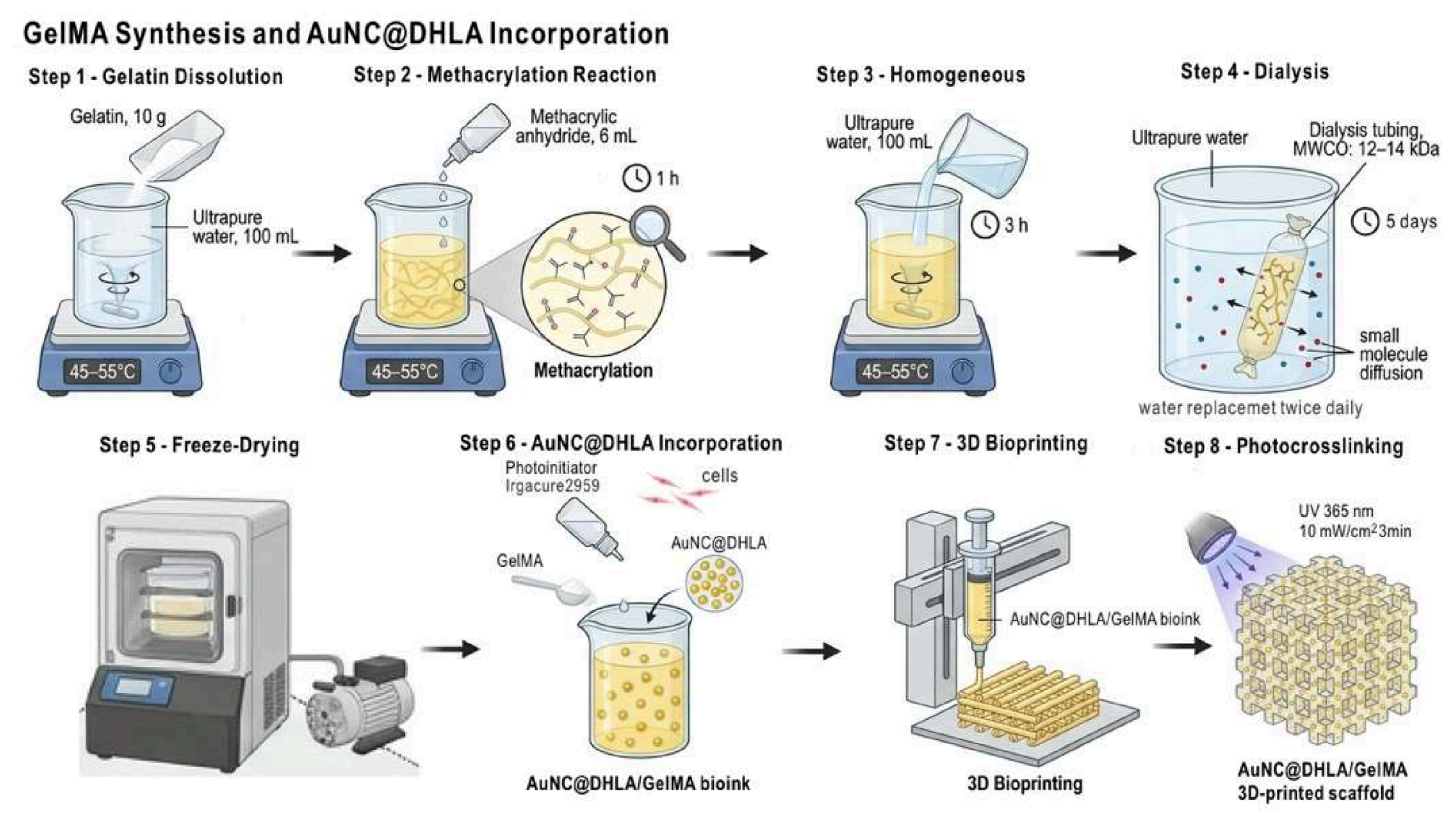
Schematic illustration of GelMA synthesis, AuNC@DHLA incorporation, and 3D bioprinting of composite scaffolds.

**Figure 1:**
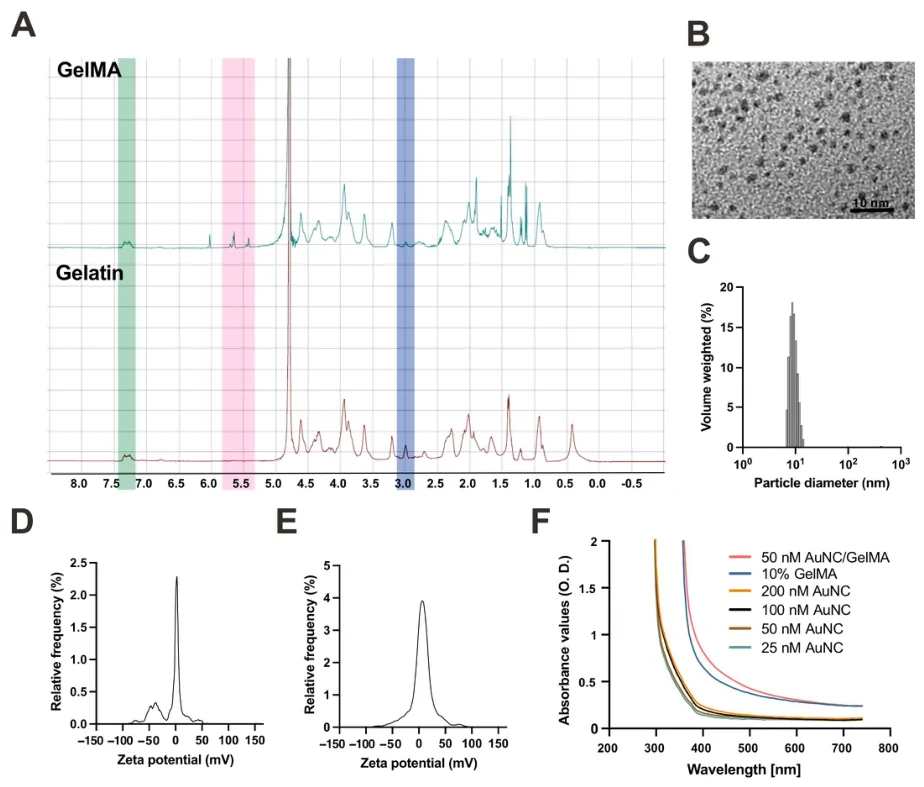
Physicochemical characterization of GelMA and AuNC@DHLA. (**A**) ^1^*H* NMR spectra of gelatin and GelMA, confirming the successful methacrylation of gelatin. (**B**) TEM image of AuNC@DHLA. Scale bar = 10 nm. (**C**) DLS analysis of AuNC@DHLA showing the hydrodynamic particle size distribution. (**D**) Zeta potential distribution of AuNC@DHLA in pure water. (**E**) Zeta potential distribution of AuNC@DHLA in 10% GelMA solution. (**F**) UV–visible absorption spectra of 10% GelMA, AuNC@DHLA at different concentrations, and 50 nM AuNC@DHLA/GelMA.

**Figure 2:**
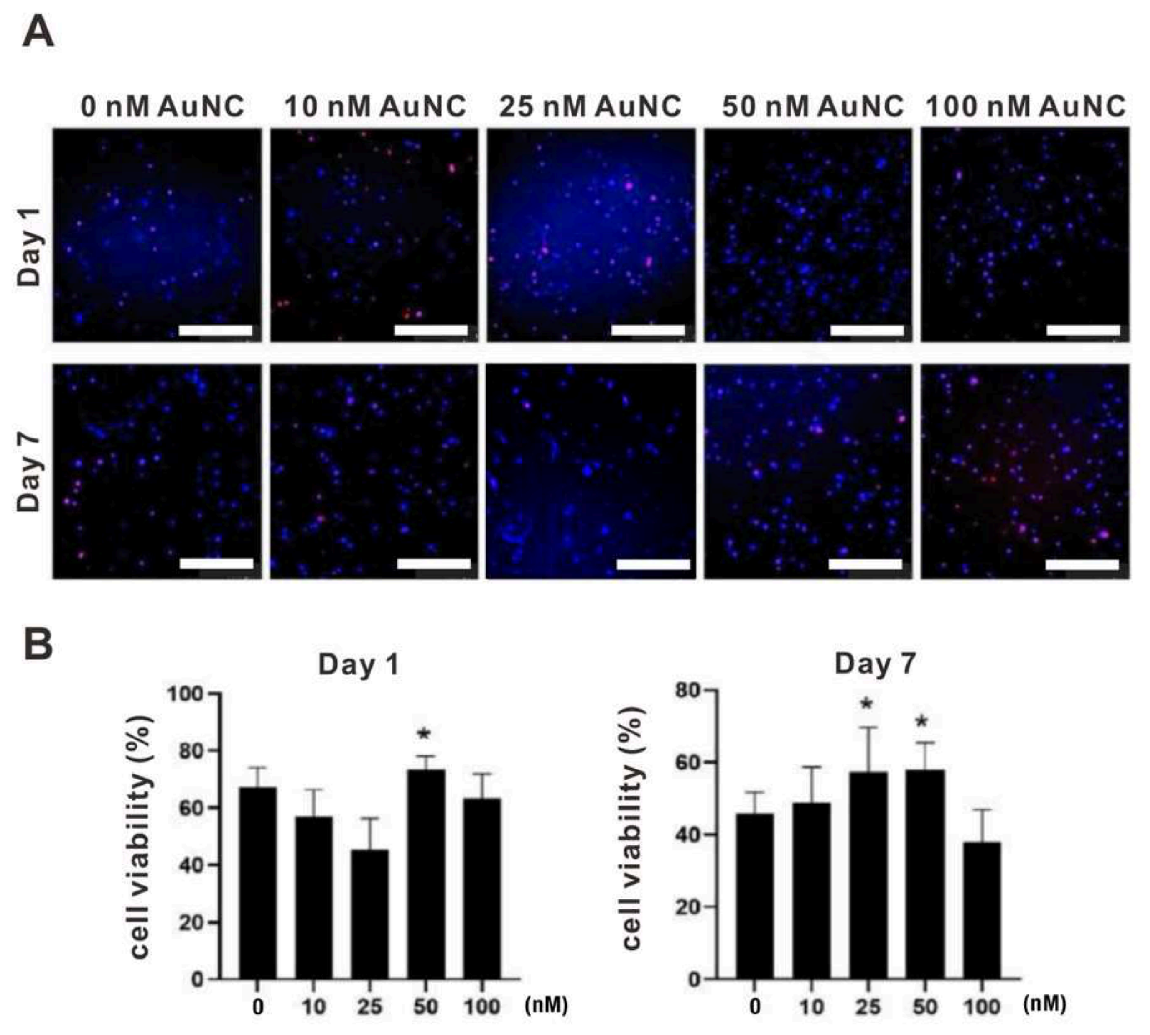
Viability of fibroblasts cultured in GelMA containing different concentrations of AuNC@DHLA. (**A**) Representative fluorescence images of fibroblasts cultured in 10% GelMA containing 0, 10, 25, 50, or 100 nM AuNC@DHLA on days 1 and 7. Nuclei were stained with DAPI (blue), and dead cells were stained with PI (red). (**B**) Quantification of cell viability on days 1 and 7. Scale bar = 200 μm. * p<0.05 compared with the 0 nM AuNC@DHLA.

**Figure 3:**
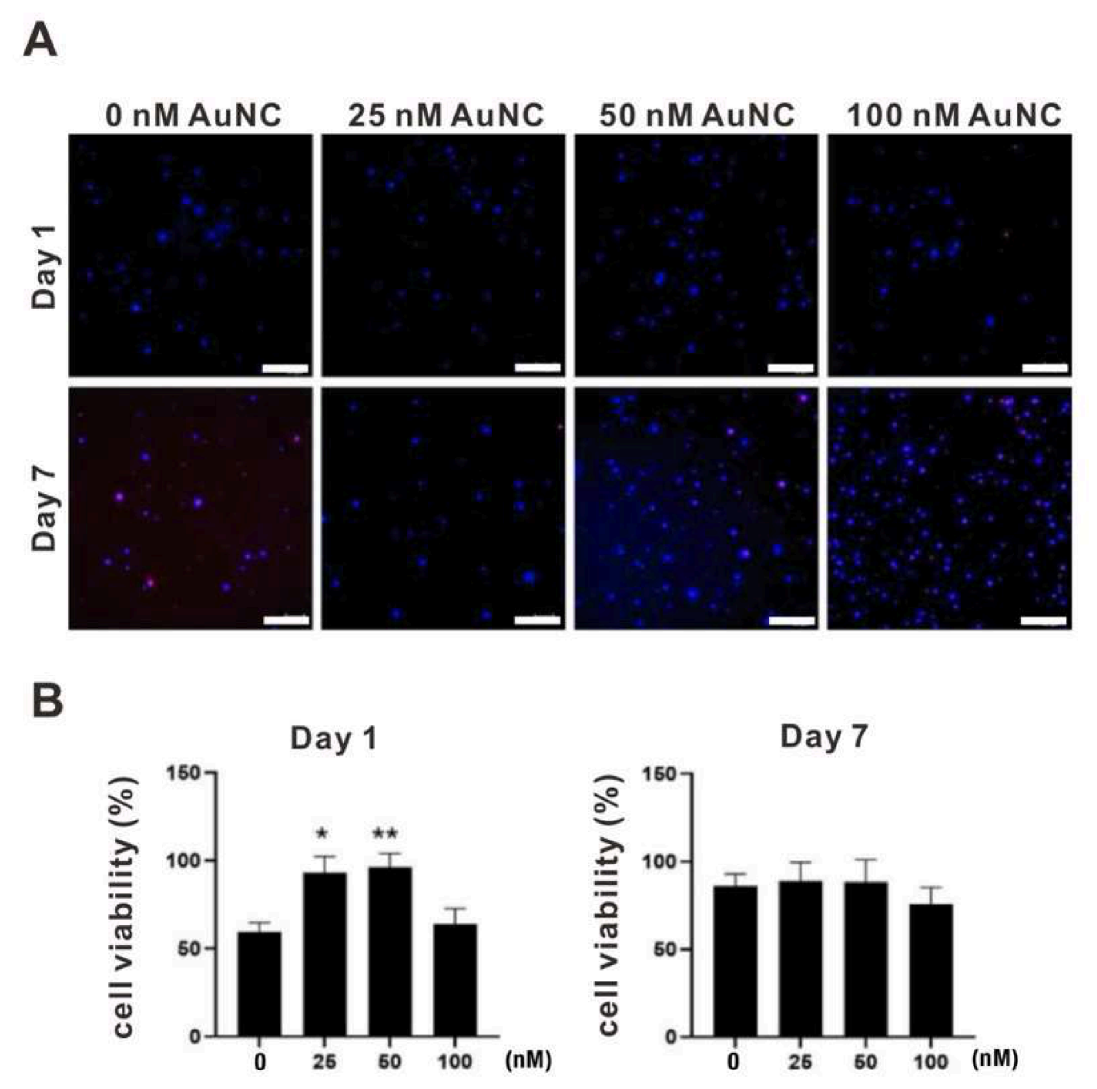
Viability of HaCaT cells cultured in GelMA containing different concentrations of AuNC@DHLA. (**A**) Representative fluorescence images of fibroblasts cultured in 10% GelMA containing 0, 25, 50, or 100 nM AuNC@DHLA on days 1 and 7. Nuclei were stained with DAPI (blue), and dead cells were stained with PI (red). (**B**) Quantification of cell viability on days 1 and 7. Scale bar = 200 μm. * *p* < 0.05 and ** *p* < 0.01 compared with the 0 nM AuNC@DHLA.

**Figure 4:**
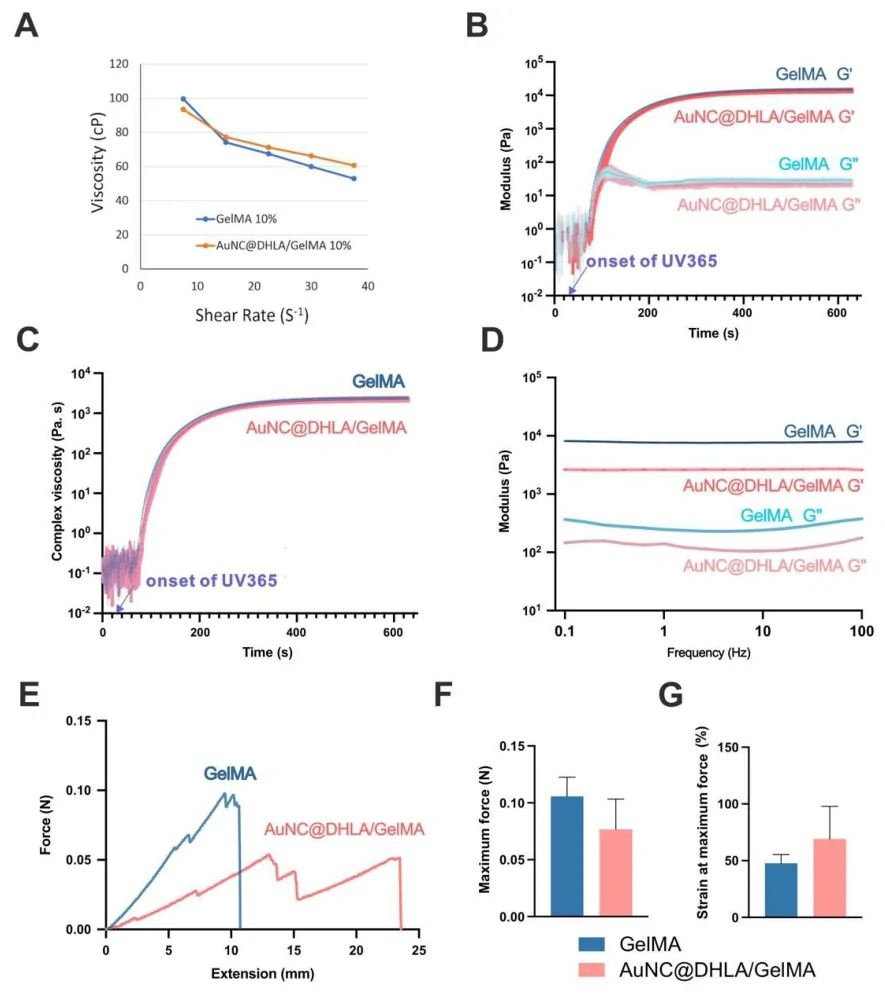
Rheological characterization of hydrogel precursors and mechanical characterization of photocrosslinked 3D-bioprinted scaffolds. (**A**) Shear rate-dependent viscosity of 10% GelMA and AuNC@DHLA/GelMA hydrogel precursor solutions. (**B**) Time sweep analysis showing the evolution of storage modulus ( $G^{\prime }$ ) and loss modulus ( $G^{\prime \prime }$ ) during UV365-induced photocrosslinking of the precursor solutions. (**C**) Changes in complex viscosity during UV365 irradiation. (**D**) Frequency sweep analysis of photocrosslinked hydrogels, showing $G^{\prime }$ and $G^{\prime \prime }$ over the tested frequency range. (**E**) Representative force–extension curves of the photocrosslinked 3D-bioprinted scaffolds. (**F**) Maximum force at failure of the scaffolds. (**G**) Strain at maximum force of the scaffolds.

**Figure 5:**
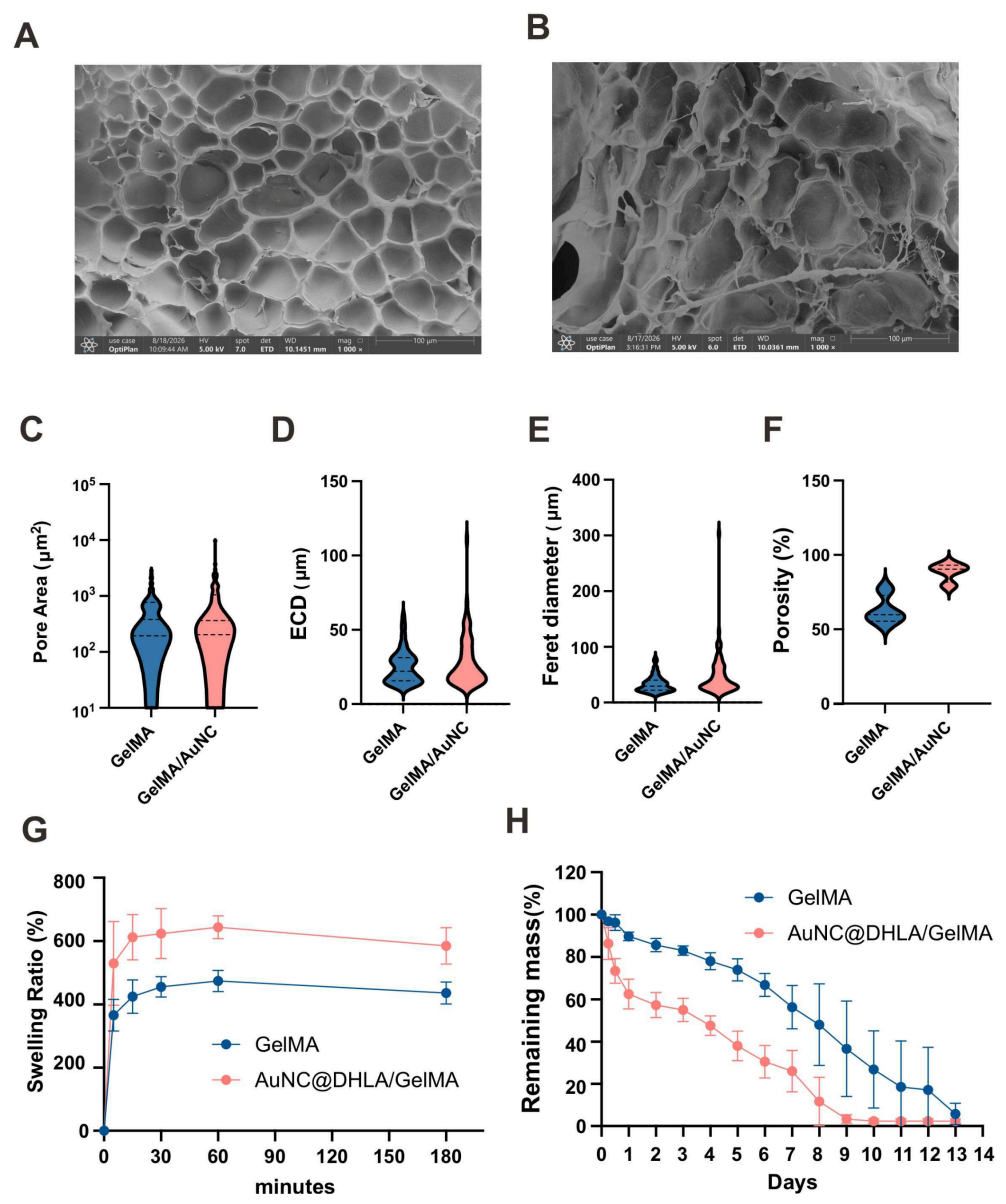
Morphological and physicochemical characterization of photocrosslinked GelMA and AuNC@DHLA/GelMA scaffolds. (**A**) Representative SEM image of the photocrosslinked GelMA scaffold. (**B**) Representative SEM image of the photocrosslinked AuNC@DHLA/GelMA scaffold. Scale bar = 100 μm. Quantification of (**C**) pore area, (**D**) equivalent circular diameter (ECD), (**E**) Feret diameter, and (**F**) porosity. (**G**) Swelling ratio of the photocrosslinked scaffolds over time. (**H**) Remaining mass of the photocrosslinked scaffolds over time.

**Figure 6:**
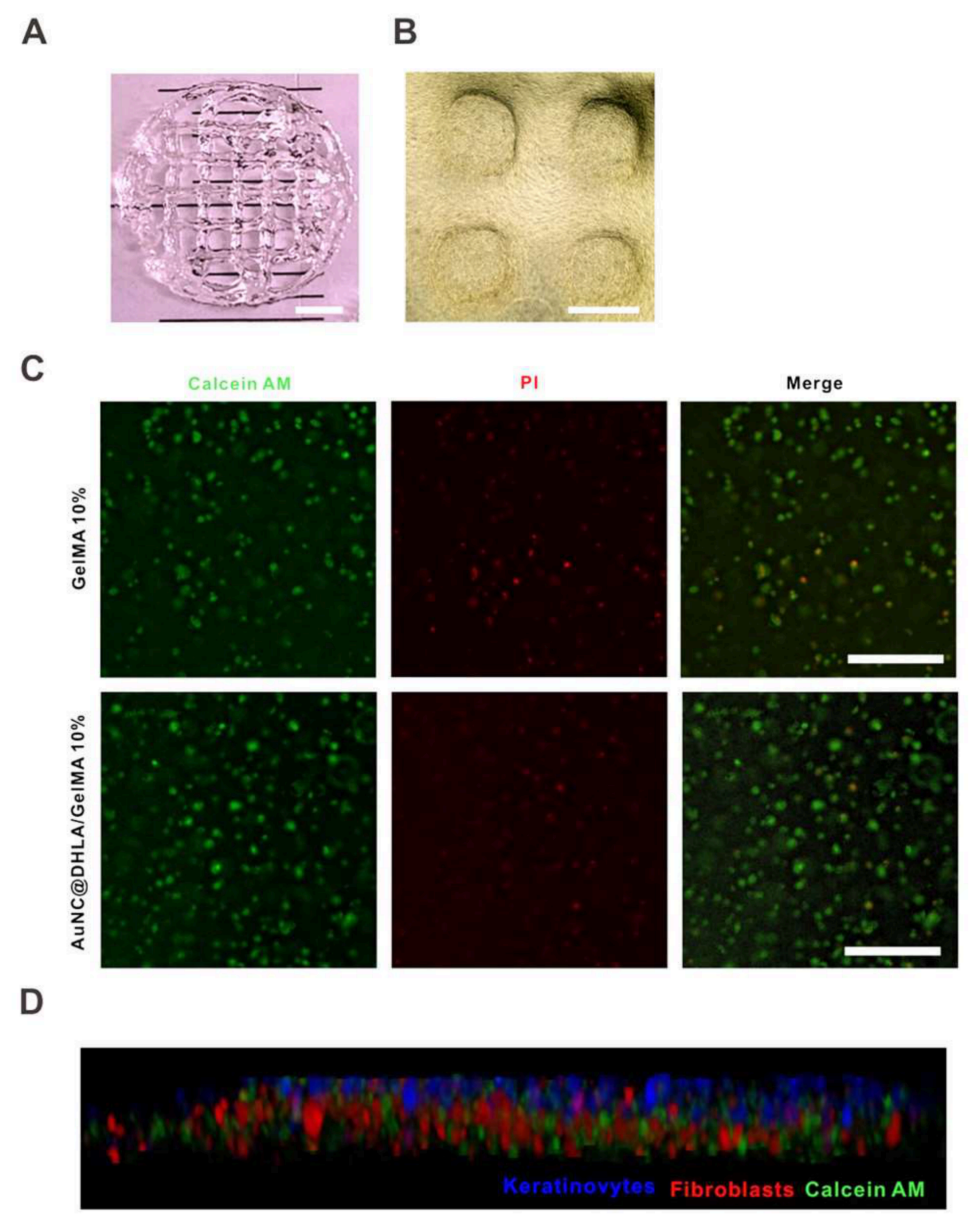
Morphological characterization and cell viability of 3D-printed cell-laden AuNC@DHLA/GelMA scaffolds. (**A**) Gross macroscopic morphology of the 3D-printed hydrogel scaffold showing a well-defined grid architecture. Scale bar = 2 mm. (**B**) Brightfield microscopic image showing the printing fidelity of the filaments and the uniform distribution of encapsulated cells within the scaffold. Scale bar = 1 mm. (**C**) Representative live/dead fluorescence images of cells encapsulated in GelMA and AuNC@DHLA/GelMA scaffolds. Live cells were stained with Calcein AM (green), and dead cells were stained with PI (red). Scale bar = 200 μm. (**D**) Three-dimensional digitally deconvoluted fluorescence cross-sectional reconstruction of the cell-laden AuNC@DHLA/GelMA scaffold, showing the stratified distribution of keratinocytes and dermal fibroblasts and the viability of encapsulated cells. Keratinocytes were labeled in blue, dermal fibroblasts in red, and live cells with Calcein AM in green.

**Figure 7:**
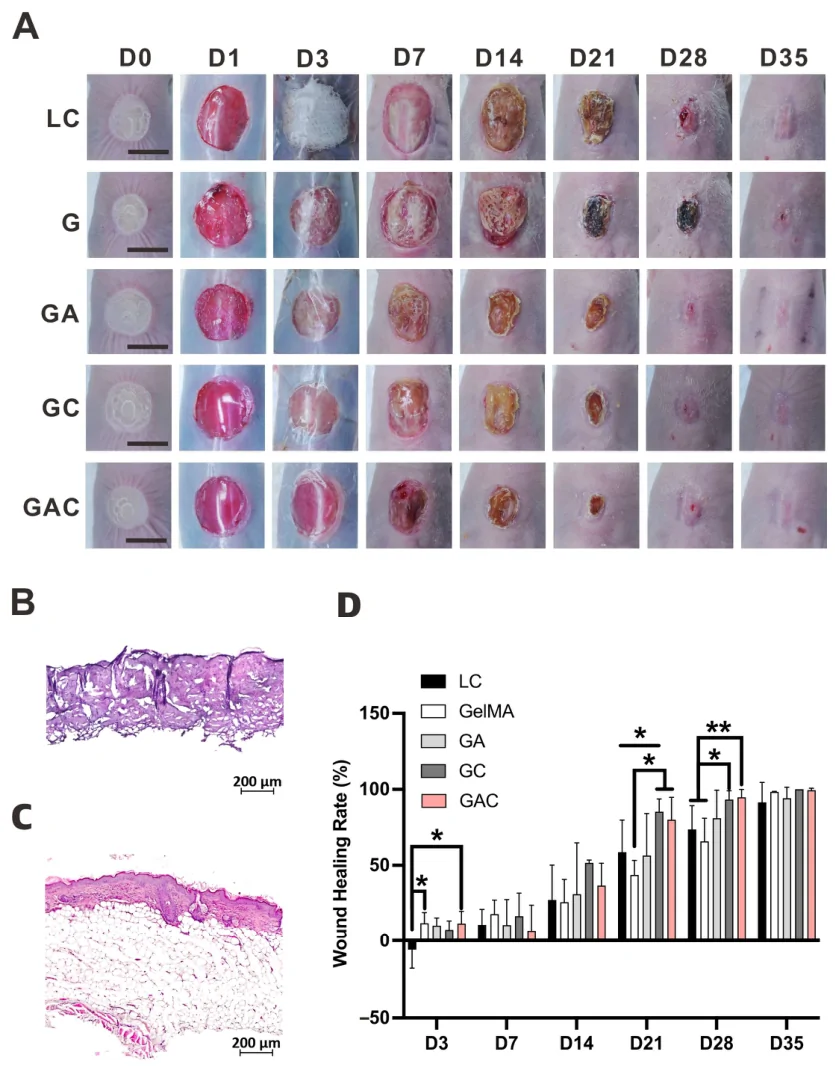
Wound healing evaluation of GelMA- and AuNC@DHLA/GelMA-based scaffolds in a burn injury model. (**A**) Representative gross images of burn wounds in the indicated groups at days 0, 1, 3, 7, 14, 21, 28, and 35. Scale bar = 1 cm. (**B**,**C**) Representative H&E staining images of burn wound tissue (**B**) and normal skin tissue (**C**). Scale bar = 200 μm. (**D**) Quantification of wound closure rate at days 3, 7, 14, 21, 28, and 35. LC, lesion control; G, GelMA; GA, AuNC@DHLA/GelMA; GC, cell-laden GelMA; and GAC, cell-laden AuNC@DHLA/GelMA scaffolds. n=5 . * p<0.05 , ** p<0.01 compared with the LC group.

**Figure 8:**
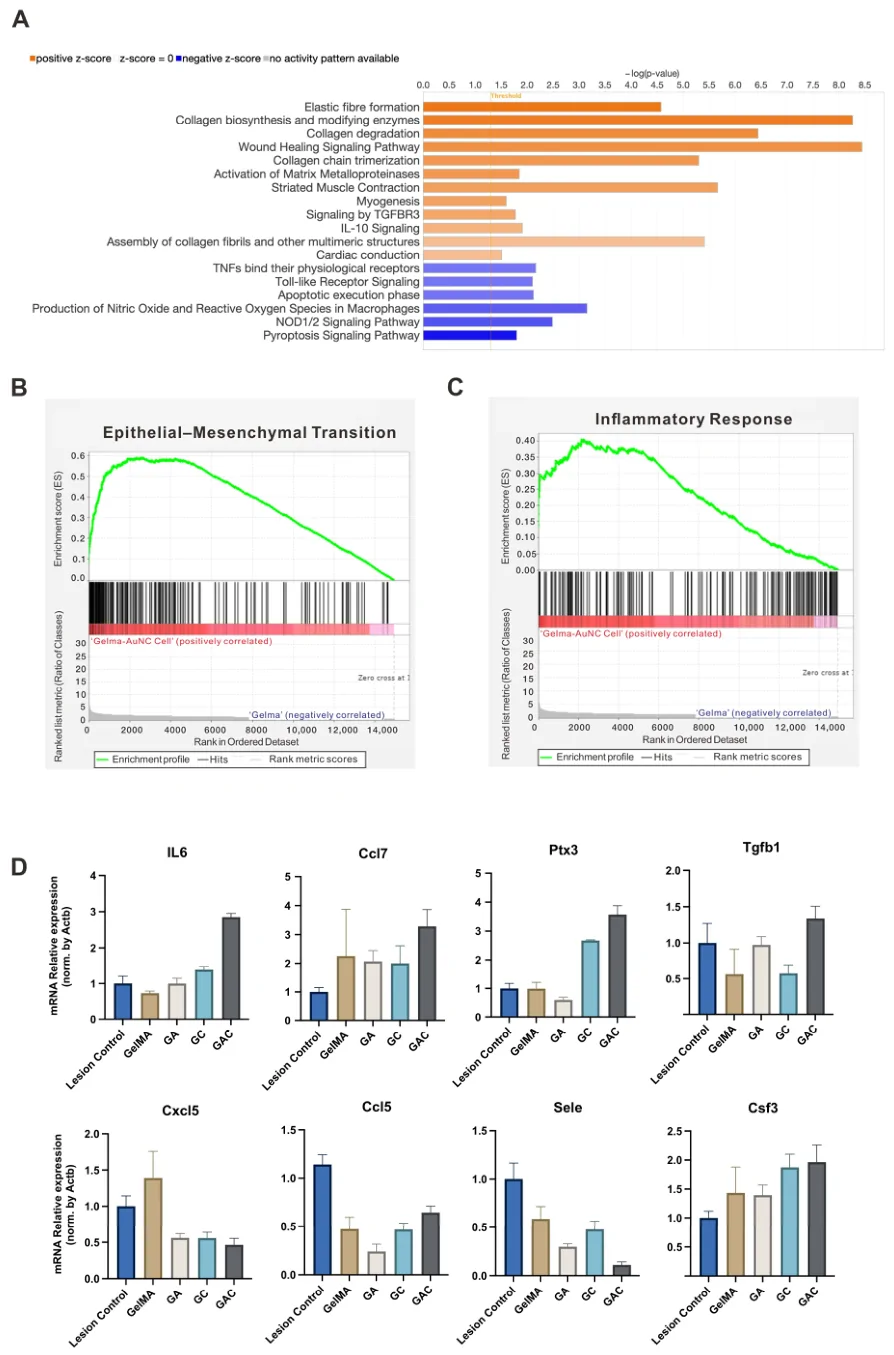
Transcriptomic profiling of burn wounds treated with the cell-laden 3D-Printed AuNC@DHLA/GelMA scaffold compared to the cell-free GelMA scaffold. (**A**–**C**) Significantly altered canonical pathway (**A**) and Enrichment plot of hallmark epithelial–mesenchymal transition and hallmark inflammatory response (**B**,**C**). (**D**) qPCR validation of representative genes associated with the EMT and inflammatory response pathways. n=3 . GA, AuNC@DHLA/GelMA; GC, cell-laden GelMA; and GAC, cell-laden AuNC@DHLA/GelMA scaffolds.

**Figure 9:**
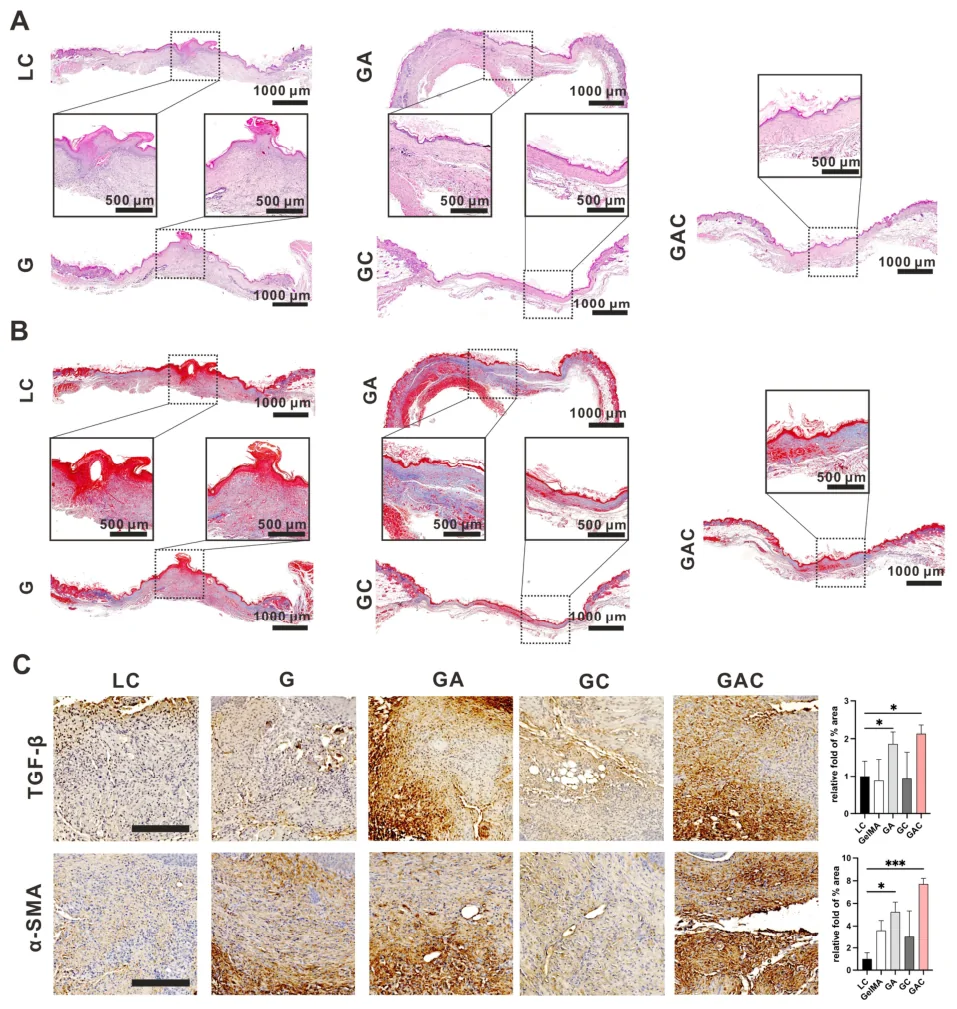
Histological and immunohistochemical characterization of wound healing in the burn injury model. (**A**,**B**) Representative H&E (**A**) and Masson’s trichrome (**B**) staining images of wound tissue on day 35, showing epidermal and granulation tissue architecture in the indicated groups. Insets show higher-magnification views of the boxed regions. n=5 . (**C**) Representative immunohistochemical staining images and quantitative analysis of TGF-β and α-SMA expression in wound tissue on day 14. Scale bar = 200 μm. n=3 . * p<0.05 , *** p<0.001 . LC, lesion control; G, GelMA; GA, AuNC@DHLA/GelMA; GC, cell-laden GelMA; and GAC, cell-laden AuNC@DHLA/GelMA scaffolds.

**Figure 10:**
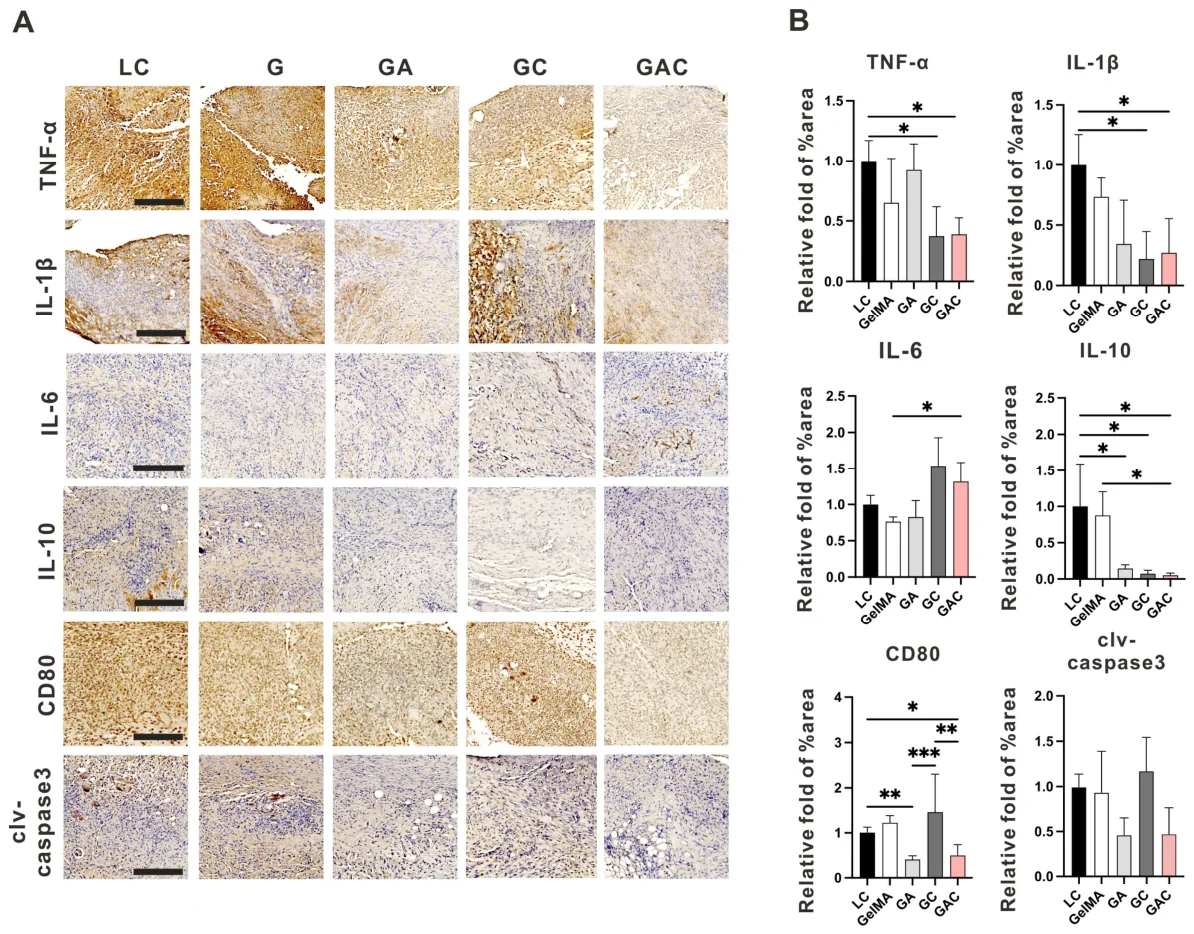
Immunohistochemical characterization of inflammatory, macrophage-associated, and apoptosis-related markers in wound tissue on day 14. (**A**) Representative immunohistochemical staining images of TNF-α, IL-1β, IL-6, IL-10, CD80, and cleaved caspase-3 (clv caspase-3) in the indicated groups. (**B**) Corresponding quantitative analysis of the positively stained area for each marker. Scale bar = 200 μm. n=3 . * p<0.05 , ** p<0.01 , *** p<0.001 . LC, lesion control; G, GelMA; GA, AuNC@DHLA/GelMA; GC, cell-laden GelMA; and GAC, cell-laden AuNC@DHLA/GelMA scaffolds.

**Table 1:** PCR primers were used in this study.

Gene Name	Primer Sequence (5′ → 3′)
*Il6*	Forward: 5′-CTGCAAGAGACTTCCATCCAG-3′ Reverse: 5′-AGTGGTATAGACAGGTCTGTTGG-3′
*Ccl7*	Forward: 5′-CCACATGCTGCTATGTCAAGA-3′ Reverse: 5′-ACACCGACTACTGGTGATCCT-3′
*Ptx3*	Forward: 5′-CGCAGGTTGTGAAACAGCAAT-3′ Reverse: 5′-GGGTTCCACTTTGTGCCATAAG-3′
*Tgfb1*	Forward: 5′-CCACCTGCAAGACCATCGAC-3′ Reverse: 5′-CTGGCGAGCCTTAGTTTGGAC-3′
*Cxcl5*	Forward: 5′-GTTCCATCTCGCCATTCATGC-3′ Reverse: 5′-GCGGCTATGACTGAGGAAGG-3′
*Ccl5*	Forward: 5′-TTTGCCTACCTCTCCCTCG-3′ Reverse: 5′-CGACTGCAAGATTGGAGCACT-3′
*Sele*	Forward: 5′-ATGAAGCCAGTGCATACTGTC-3′ Reverse: 5′-CGGTGAATGTTTCAGATTGGAGT-3′
*Csf3*	Forward: 5′-TTCCCTGAGTGGCTGCTCTA-3′ Reverse: 5′-TGAAGCAAGTCCAAGGTGGG-3′

## Data Availability

The datasets generated and/or analyzed during this study are available from the corresponding author upon reasonable request. Additionally, our RNA-seq data has been deposited in the NCBI Gene Expression Omnibus under accession number GSE330443.

## Supplementary Materials

The following supporting information can be downloaded at: https://www.mdpi.com/article/10.3390/polym18192331/s1, Table S1: Hallmark Epithelial Mesenchymal Transition. Table S2: Hallmark Inflammatory Response.

## Abbreviations

The following abbreviations are used in this manuscript:

3D	Three-dimensional
ECM	extracellular matrix
ROS	reactive oxygen species
AuNCs	Gold nanoparticles
DHLA	dihydrolipoic acid
ALA	alpha-lipoic acid
GelMA	Gelatin methacryloyl
DEGs	Differentially expressed genes
FDR	false discovery rate
IPA	Ingenuity Pathway Analysis
LC	lesion control
DAB	3,3′-diaminobenzidine
SD	standard deviation
ANOVA	analysis of variance
DAPI	4′,6-diamidino-2-phenylindole
MMPs	Matrix Metalloproteinases
GSEA	Gene Set Enrichment Analysis
Il6	interleukin-6
Ptx3	pentraxin 3
Cxcl5	C-X-C motif chemokine ligand 5
TGF-β	transforming growth factor-beta 1
α-SMA	alpha-smooth muscle actin
EMT	epithelial–mesenchymal transition
TLR	Toll-like Receptor
