# Supplementary material for: A Bioactive 3D-Bioprinted AuNC@DHLA/GelMA Hydrogel Containing Skin Cells Promotes Burn Wound Healing and Tissue Regeneration
Source: Polymers (Basel). 2026 Sep 24;18(19):2331. doi: 10.3390/polym18192331 (PMC13666594; doi:10.3390/polym18192331)
Supplement: Supplementary file 1 [file polymers-18-02331-s001.zip › polymers-4434188-SM.pdf]

Table S1. HALLMARK_EPITHELIAL_MESENCHYMAL_TRANSITION.

NAME	SYMBOL	TITLE	RANK IN GENE LIST	RANK METRIC SCORE	RUNNING ES	CORE ENRICHMENT
row_0	Il6	NA	0	32.460330963134800	0.08794254	Yes
row_1	Ptx3	NA	6	8.52866268157959	0.1107094	Yes
row_2	Cxcl5	NA	7	8.527896881103520	0.13381344	Yes
row_3	Bdnf	NA	22	5.749969005584720	0.14844151	Yes
row_4	Mgp	NA	27	5.45810079574585	0.16295737	Yes
row_5	Igfbp4	NA	42	4.678469657897950	0.1746825	Yes
row_6	Ccn1	NA	61	4.112528324127200	0.18460295	Yes
row_7	Fmod	NA	73	3.9416251182556200	0.19453536	Yes
row_8	Timp1	NA	85	3.7123117446899400	0.2038465	Yes
row_9	Gas1	NA	96	3.5922634601593000	0.21290025	Yes
row_10	Pcolce	NA	98	3.5760598182678200	0.22252077	Yes
row_11	Bgn	NA	132	3.322547435760500	0.22928321	Yes
row_12	Gem	NA	138	3.2525079250335700	0.23775575	Yes
row_13	Pdlim4	NA	167	3.1166210174560500	0.24429955	Yes
row_14	Mmp3	NA	170	3.106043815612790	0.25257882	Yes
row_15	Col1a1	NA	183	3.037071704864500	0.25999275	Yes
row_16	Cthrc1	NA	185	3.035715341567990	0.26814935	Yes
row_17	Mfap5	NA	191	3.023726224899290	0.27600205	Yes
row_18	Col8a2	NA	203	2.9982688426971400	0.2833787	Yes
row_19	Gpx7	NA	210	2.967189311981200	0.29101038	Yes
row_20	Igfbp2	NA	230	2.911231756210330	0.2976084	Yes
row_21	Loxl1	NA	236	2.9021661281585700	0.3051318	Yes
row_22	Eln	NA	238	2.899625539779660	0.31291968	Yes
row_23	Efemp2	NA	260	2.819808006286620	0.3191343	Yes
row_24	Sparc	NA	295	2.7424936294555700	0.3242574	Yes
row_25	Crlf1	NA	331	2.648470640182500	0.3290579	Yes
row_26	Fbln1	NA	337	2.6387312412262000	0.33586758	Yes
row_27	Lgals1	NA	344	2.6302099227905300	0.3425863	Yes
row_28	Vcam1	NA	345	2.628695011138920	0.34970805	Yes
row_29	Col1a2	NA	356	2.6051628589630100	0.35608754	Yes
row_30	Fbln5	NA	381	2.5607101917266800	0.36139664	Yes
row_31	Serpine2	NA	390	2.5460145473480200	0.36775157	Yes
row_32	Serpine1	NA	405	2.5221993923187300	0.37363487	Yes
row_33	Thy1	NA	437	2.468567371368410	0.37821937	Yes
row_34	Myl9	NA	445	2.4585068225860600	0.38440508	Yes
row_35	Ccn2	NA	471	2.427600383758550	0.3892857	Yes
row_36	Cxcl12	NA	482	2.403733015060430	0.39511946	Yes
row_37	Col3a1	NA	492	2.391493797302250	0.4009879	Yes
row_38	Col16a1	NA	505	2.371917963027950	0.40659976	Yes
row_39	Vegfc	NA	514	2.3612723350524900	0.4124542	Yes
row_40	Sfrp1	NA	529	2.346426010131840	0.41786125	Yes
row_41	Bmp1	NA	539	2.338656425476070	0.42358655	Yes
row_42	Areg	NA	550	2.3249082565307600	0.42920676	Yes
row_43	Tnfrsf12a	NA	566	2.3098769187927200	0.43444696	Yes
row_44	Tgfbr3	NA	606	2.2711474895477300	0.4379538	Yes
row_45	Fbln2	NA	630	2.2512271404266400	0.4424923	Yes
row_46	Mest	NA	633	2.248591184616090	0.44844857	Yes
row_47	Nnmt	NA	635	2.2470362186431900	0.45446846	Yes
row_48	Mmp2	NA	637	2.242039442062380	0.4604748	Yes
row_49	Matn2	NA	658	2.217526912689210	0.46512556	Yes
row_50	P3h1	NA	659	2.216012477874760	0.47112924	Yes
row_51	Pvr	NA	689	2.1920087337493900	0.4751002	Yes
row_52	Aplp1	NA	717	2.1697998046875	0.4791467	Yes
row_53	Abi3bp	NA	728	2.159895896911620	0.48431984	Yes
row_54	Tagln	NA	735	2.1542584896087600	0.4897491	Yes
row_55	Msx1	NA	740	2.150637626647950	0.4953043	Yes
row_56	Nid2	NA	748	2.146717071533200	0.5006453	Yes
row_57	Tfpi2	NA	801	2.1122958660125700	0.5028397	Yes
row_58	Dcn	NA	818	2.0986697673797600	0.5074398	Yes
row_59	Tpm1	NA	848	2.0867362022399900	0.51112556	Yes
row_60	Col6a2	NA	858	2.079310178756710	0.5161482	Yes
row_61	Serpinh1	NA	936	2.0328526496887200	0.5164311	Yes
row_62	Slit3	NA	953	2.022785186767580	0.5208257	Yes
row_63	Lum	NA	1028	1.9789624214172400	0.5211661	Yes
row_64	Vim	NA	1041	1.9751696586608900	0.5257031	Yes
row_65	Col5a1	NA	1094	1.9530287981033300	0.527466	Yes
row_66	Thbs2	NA	1106	1.9467554092407200	0.5319938	Yes
row_67	Edil3	NA	1130	1.9323463439941400	0.53566843	Yes
row_68	Col5a2	NA	1149	1.9211419820785500	0.5396519	Yes
row_69	Ppib	NA	1152	1.9192570447921800	0.5447159	Yes
row_70	Itga5	NA	1202	1.8997052907943700	0.54653794	Yes
row_71	Plod1	NA	1213	1.8962222337722800	0.5509967	Yes
row_72	Pmp22	NA	1374	1.840417742729190	0.5451265	Yes
row_73	Tpm2	NA	1391	1.8366469144821200	0.5490168	Yes
row_74	Plod3	NA	1401	1.8340487480163600	0.553375	Yes
row_75	Pmepa1	NA	1441	1.8206707239151	0.5556614	Yes
row_76	Prrx1	NA	1524	1.7949275970459000	0.5549604	Yes
row_77	Slit2	NA	1557	1.7819644212722800	0.5576169	Yes
row_78	Lox	NA	1618	1.7642053365707400	0.55832547	Yes
row_79	Tnc	NA	1654	1.7531324625015300	0.5607003	Yes
row_80	Tgfb1	NA	1658	1.7525525093078600	0.5652448	Yes
row_81	Foxc2	NA	1693	1.7424148321151700	0.5676584	Yes
row_82	Sfrp4	NA	1778	1.7202889919281000	0.5666195	Yes
row_83	Ecm2	NA	1780	1.7197123765945400	0.5712108	Yes
row_84	Fstl1	NA	1835	1.7044923305511500	0.57216465	Yes
row_85	Grem1	NA	1878	1.6944444179534900	0.5739055	Yes
row_86	Postn	NA	1881	1.6937869787216200	0.57835865	Yes
row_87	Emp3	NA	1900	1.6896326541900600	0.5817149	Yes
row_88	Col4a2	NA	1913	1.6848032474517800	0.58546525	Yes
row_89	Fstl3	NA	2025	1.659082055091860	0.5824285	Yes
row_90	Col12a1	NA	2066	1.6505078077316300	0.5841861	Yes
row_91	Htra1	NA	2155	1.6305031776428200	0.58263254	Yes
row_92	Lrp1	NA	2208	1.6194486618042000	0.5834917	Yes
row_93	Gpc1	NA	2269	1.6049246788024900	0.58376867	Yes
row_94	Col4a1	NA	2312	1.5979853868484500	0.58524823	Yes
row_95	Adam12	NA	2331	1.5942432880401600	0.58834606	Yes
row_96	Igfbp3	NA	2433	1.5762200355529800	0.5857634	Yes
row_97	Itgb5	NA	2496	1.5663248300552400	0.5858001	Yes
row_98	Mmp14	NA	2538	1.5569449663162200	0.5872363	Yes
row_99	Sgcd	NA	2577	1.5501484870910600	0.58885765	Yes
row_100	Cdh11	NA	2644	1.538556694984440	0.5885477	No
row_101	Pdgfrb	NA	2813	1.5112721920013400	0.581243	No
row_102	Cdh2	NA	2989	1.4828752279281600	0.5733864	No
row_103	Fzd8	NA	3061	1.4692795276641800	0.5725495	No
row_104	Anpep	NA	3071	1.467980980873110	0.575916	No
row_105	Acta2	NA	3217	1.44477117061615	0.56999165	No
row_106	Sdc4	NA	3251	1.439754843711850	0.5716532	No
row_107	Plod2	NA	3258	1.4390795230865500	0.5751449	No
row_108	Fbn2	NA	3288	1.4355052709579500	0.5770663	No
row_109	Fbn1	NA	3393	1.421537160873410	0.573861	No
row_110	Thbs1	NA	3441	1.4127782583236700	0.5744995	No
row_111	Sgcg	NA	3452	1.4120349884033200	0.5776465	No
row_112	Mylk	NA	3496	1.4049512147903400	0.5785352	No
row_113	Col5a3	NA	3546	1.400181531906130	0.5790039	No
row_114	Lrrc15	NA	3607	1.392656683921810	0.5787058	No
row_115	Sgcb	NA	3649	1.3852959871292100	0.579677	No
row_116	Comp	NA	3744	1.3706996440887500	0.5770124	No
row_117	Fap	NA	3799	1.3644529581069900	0.5770451	No
row_118	Dpysl3	NA	3801	1.3642034530639600	0.58067316	No
row_119	Tgfbi	NA	3919	1.3497989177703900	0.5763914	No
row_120	Cdh6	NA	3934	1.3480788469314600	0.57909375	No
row_121	Itgb3	NA	3966	1.343854546546940	0.58063114	No
row_122	Cap2	NA	4050	1.3341768980026200	0.57861406	No
row_123	Vcan	NA	4129	1.3246114253997800	0.57691026	No
row_124	Capg	NA	4165	1.320764183998110	0.57811373	No
row_125	Fn1	NA	4194	1.3182021379470800	0.57978517	No
row_126	Fuca1	NA	4216	1.314541220664980	0.5819217	No
row_127	Col11a1	NA	4240	1.3120671510696400	0.58391577	No
row_128	Colgalt1	NA	4363	1.2970376014709500	0.57915187	No
row_129	Dab2	NA	4481	1.2847256660461400	0.5746938	No
row_130	Lama2	NA	4495	1.2834372520446800	0.57728887	No
row_131	Lamc1	NA	4599	1.2705036401748700	0.5737422	No
row_132	Glipr1	NA	4615	1.26932692527771	0.57616335	No
row_133	Fermt2	NA	4704	1.2593498229980500	0.5736042	No
row_134	Col7a1	NA	4849	1.2424111366272000	0.5671995	No
row_135	Col6a3	NA	4881	1.2385305166244500	0.56845164	No
row_136	Calu	NA	5042	1.2228823900222800	0.5609084	No
row_137	Ecm1	NA	5084	1.2188777923584000	0.56142867	No
row_138	Qsox1	NA	5389	1.187036156654360	0.5440177	No
row_139	Tgm2	NA	5450	1.1804136037826500	0.5431446	No
row_140	Pfn2	NA	6173	1.113900899887090	0.4971734	No
row_141	Rhob	NA	6184	1.1129124164581300	0.49951002	No
row_142	Pcolce2	NA	6362	1.0986073017120400	0.49047664	No
row_143	Itgb1	NA	6599	1.079017996788030	0.47738692	No
row_144	Cald1	NA	6722	1.0695796012878400	0.47200674	No
row_145	Fgf2	NA	6745	1.0677603483200100	0.47340682	No
row_146	Tpm4	NA	7149	1.0348124504089400	0.44886607	No
row_147	Magee1	NA	7285	1.022216796875	0.4424755	No
row_148	Tnfrsf11b	NA	7701	0.9927372336387630	0.41700655	No
row_149	Sntb1	NA	7735	0.9904253482818600	0.41745073	No
row_150	Slc6a8	NA	7943	0.9740746021270750	0.4060444	No
row_151	Wipf1	NA	8086	0.962092936038971	0.399016	No
row_152	Mcm7	NA	8145	0.9572436809539800	0.39767396	No
row_153	Flna	NA	8469	0.9348734617233280	0.37829062	No
row_154	Pthlh	NA	8678	0.91951984167099	0.36666864	No
row_155	Sdc1	NA	8752	0.913891613483429	0.3641914	No
row_156	Id2	NA	9534	0.8564894795417790	0.31351957	No
row_157	Copa	NA	9550	0.8555657267570500	0.31481972	No
row_158	Cadm1	NA	9646	0.8493329286575320	0.31067482	No
row_159	Jun	NA	10413	0.7942408323287960	0.26085213	No
row_160	Snai2	NA	10578	0.7836852073669430	0.2518476	No
row_161	Eno2	NA	10612	0.7818139791488650	0.25172663	No
row_162	Itgav	NA	10698	0.7756044864654540	0.24806051	No
row_163	Inhba	NA	10801	0.7688503265380860	0.24322261	No
row_164	Fas	NA	11064	0.7503440380096440	0.2274783	No
row_165	Rgs4	NA	11105	0.7478393316268920	0.2267903	No
row_166	Plaur	NA	11291	0.7349716424942020	0.21622892	No
row_167	Wnt5a	NA	11477	0.7213371396064760	0.2056306	No
row_168	Itga2	NA	11712	0.7049188613891600	0.19166306	No
row_169	Gja1	NA	12016	0.6837696433067320	0.17295645	No
row_170	Dst	NA	12388	0.6554993987083440	0.14955933	No
row_171	Tnfaip3	NA	12424	0.6531640291213990	0.1489541	No
row_172	Gadd45a	NA	12581	0.6394875645637510	0.14010173	No
row_173	Vegfa	NA	12633	0.6343561410903930	0.1383599	No
row_174	Cd44	NA	12685	0.6303060054779050	0.1366071	No
row_175	Notch2	NA	12687	0.6301193237304690	0.13824639	No
row_176	Sat1	NA	12778	0.6222048401832580	0.13382542	No
row_177	Nt5e	NA	13118	0.5909059047698980	0.11242456	No
row_178	Spp1	NA	13199	0.5823869109153750	0.108574234	No
row_179	Basp1	NA	13355	0.5666038393974300	0.09959227	No
row_180	Timp3	NA	13480	0.554108202457428	0.09267985	No
row_181	Lama1	NA	14373	0.41267719864845300	0.03327407	No
row_182	Lamc2	NA	14437	0.3945996165275570	0.030068466	No
row_183	Gadd45b	NA	14589	0.3391830027103420	0.020741768	No
row_184	Lama3	NA	14604	0.33338406682014500	0.020695059	No
row_185	Il15	NA	14631	0.32558268308639500	0.01981299	No

Table S2. HALLMARK_INFLAMMATORY_RESPONSE.

NAME	SYMBOL	TITLE	RANK IN GENE LIST	RANK METRIC SCORE	RUNNING ES	CORE ENRICHMENT
row_0	Il6	NA	0	32.460330963134800	0.13754174	Yes
row_1	Cxcl5	NA	7	8.527896881103520	0.17326967	Yes
row_2	Rgs16	NA	12	6.643903732299810	0.20115025	Yes
row_3	Bdkrb1	NA	13	6.640013694763180	0.2292855	Yes
row_4	Csf3	NA	43	4.677713871002200	0.24714032	Yes
row_5	Tnfaip6	NA	62	4.110343933105470	0.2633367	Yes
row_6	Sele	NA	68	4.04927396774292	0.28015545	Yes
row_7	Timp1	NA	85	3.7123117446899400	0.29480082	Yes
row_8	Ccl5	NA	302	2.714146137237550	0.2916602	Yes
row_9	Ccr7	NA	369	2.588730573654180	0.29815555	Yes
row_10	Serpine1	NA	405	2.5221993923187300	0.30647027	Yes
row_11	Ccl7	NA	632	2.250276565551760	0.3006863	Yes
row_12	Pvr	NA	689	2.1920087337493900	0.30617848	Yes
row_13	Scn1b	NA	692	2.18969464302063	0.31532115	Yes
row_14	Il1r1	NA	807	2.1090309619903600	0.31653035	Yes
row_15	Sphk1	NA	866	2.076406478881840	0.32139716	Yes
row_16	Has2	NA	869	2.0756542682647700	0.3300566	Yes
row_17	Gpc3	NA	873	2.0733861923217800	0.33863866	Yes
row_18	Lpar1	NA	1033	1.97780179977417	0.33624157	Yes
row_19	Bst2	NA	1098	1.9517204761505100	0.34017336	Yes
row_20	Itga5	NA	1202	1.8997052907943700	0.3412412	Yes
row_21	Tnfrsf9	NA	1247	1.8847529888153100	0.3462449	Yes
row_22	Myc	NA	1322	1.8584264516830400	0.34910354	Yes
row_23	Ptger4	NA	1442	1.8205231428146400	0.34875134	Yes
row_24	Chst2	NA	1491	1.8026928901672400	0.35313618	Yes
row_25	Gpr183	NA	1528	1.7942163944244400	0.3582985	Yes
row_26	Ebi3	NA	1551	1.7834128141403200	0.364364	Yes
row_27	Scarf1	NA	1600	1.7709790468215900	0.36861446	Yes
row_28	Tpbg	NA	1636	1.7579375505447400	0.37369084	Yes
row_29	Hbegf	NA	1667	1.7504935264587400	0.3790746	Yes
row_30	Tapbp	NA	1704	1.7396087646484400	0.38400552	Yes
row_31	Aplnr	NA	1856	1.6988544464111300	0.38096875	Yes
row_32	Emp3	NA	1900	1.6896326541900600	0.38521343	Yes
row_33	Hpn	NA	1983	1.667739987373350	0.38672185	Yes
row_34	Selenos	NA	1994	1.664974331855770	0.3930989	Yes
row_35	Cd40	NA	2115	1.6388118267059300	0.39190897	Yes
row_36	Tnfsf9	NA	2138	1.6335139274597200	0.3973393	Yes
row_37	Il4ra	NA	2144	1.6321911811828600	0.40391636	Yes
row_38	Adora2b	NA	2245	1.611334204673770	0.40396565	Yes
row_39	Ly6e	NA	2428	1.5772112607955900	0.39831218	No
row_40	Mmp14	NA	2538	1.5569449663162200	0.397521	No
row_41	Ptgir	NA	2889	1.5007250308990500	0.38015592	No
row_42	Il15ra	NA	2938	1.4928038120269800	0.38322768	No
row_43	Osmr	NA	3080	1.4657528400421100	0.37988102	No
row_44	Calcrl	NA	3111	1.4623154401779200	0.3840437	No
row_45	Ifngr2	NA	3381	1.4226727485656700	0.3718383	No
row_46	Pik3r5	NA	3413	1.4171233177185100	0.37574172	No
row_47	Sri	NA	3414	1.4168692827224700	0.3817453	No
row_48	P2rx4	NA	3466	1.409342646598820	0.3842601	No
row_49	Slc7a2	NA	3762	1.3687835931778000	0.370064	No
row_50	Rgs1	NA	3903	1.351220965385440	0.36629984	No
row_51	Itgb3	NA	3966	1.343854546546940	0.3677915	No
row_52	Rela	NA	3994	1.3395920991897600	0.37163752	No
row_53	Cx3cl1	NA	3999	1.3389081954956100	0.37703964	No
row_54	Axl	NA	4093	1.329201340675350	0.376368	No
row_55	Cd82	NA	4144	1.3230165243148800	0.37858474	No
row_56	Pde4b	NA	4245	1.3117859363555900	0.3773648	No
row_57	Gpr132	NA	4476	1.2851171493530300	0.36722007	No
row_58	P2ry2	NA	4487	1.2841358184814500	0.37198344	No
row_59	Pdpn	NA	4553	1.277706265449520	0.37299147	No
row_60	Btg2	NA	4605	1.270126461982730	0.37491637	No
row_61	Icam1	NA	4670	1.2632508277893100	0.37593094	No
row_62	Tacr1	NA	4744	1.254538655281070	0.37629855	No
row_63	Adrm1	NA	4890	1.2379506826400800	0.37171552	No
row_64	Slc4a4	NA	5023	1.2243996858596800	0.36795625	No
row_65	Ereg	NA	5139	1.2116607427597000	0.3652953	No
row_66	Lck	NA	5177	1.2076737880706800	0.3679045	No
row_67	Nmi	NA	5544	1.1711541414260900	0.34805846	No
row_68	Rhog	NA	5669	1.1598063707351700	0.34456775	No
row_69	Slc11a2	NA	5695	1.1576215028762800	0.3477783	No
row_70	Irf7	NA	6596	1.0790629386901900	0.29134598	No
row_71	Cxcl9	NA	6695	1.072214961051940	0.28924647	No
row_72	Ccl22	NA	7318	1.0203582048416100	0.25140905	No
row_73	Gabbr1	NA	7434	1.0106006860733000	0.24789616	No
row_74	Edn1	NA	7460	1.0092204809188800	0.25047788	No
row_75	Il2rb	NA	7615	0.9993261694908140	0.2442737	No
row_76	Lif	NA	7638	0.997048556804657	0.2470072	No
row_77	Irf1	NA	7982	0.9709784984588620	0.22787195	No
row_78	Acvr1b	NA	8078	0.962622880935669	0.22551143	No
row_79	Ffar2	NA	8168	0.9558389782905580	0.22352886	No
row_80	P2rx7	NA	8403	0.9391319751739500	0.211647	No
row_81	Nfkb1	NA	8570	0.9271126389503480	0.20432344	No
row_82	F3	NA	8599	0.9246155023574830	0.20634332	No
row_83	Adgre1	NA	8911	0.9016305208206180	0.18908328	No
row_84	Cdkn1a	NA	9172	0.8821884989738460	0.17519778	No
row_85	Ccl24	NA	9202	0.880378007888794	0.17696244	No
row_86	Cd48	NA	9222	0.8785480260849000	0.17939717	No
row_87	Rtp4	NA	9305	0.8732044696807860	0.17753895	No
row_88	Ripk2	NA	9537	0.8562967777252200	0.16550943	No
row_89	Nfkbia	NA	9574	0.8540378212928770	0.166688	No
row_90	Tlr3	NA	9673	0.8477344512939450	0.16363733	No
row_91	Pcdh7	NA	9686	0.8468136787414550	0.16641207	No
row_92	Ldlr	NA	10091	0.8190965056419370	0.14249851	No
row_93	Lyn	NA	10365	0.7969255447387700	0.12737055	No
row_94	Ifnar1	NA	10398	0.7949835658073430	0.12857002	No
row_95	Icosl	NA	10541	0.7855148911476140	0.12227327	No
row_96	Abca1	NA	10579	0.7836843729019170	0.12308595	No
row_97	Il10ra	NA	10655	0.778667688369751	0.12130163	No
row_98	Psen1	NA	10761	0.7713729739189150	0.11745292	No
row_99	Inhba	NA	10801	0.7688503265380860	0.118067175	No
row_100	Stab1	NA	10871	0.7630869746208190	0.116623536	No
row_101	Hif1a	NA	11222	0.7395329475402830	0.09603312	No
row_102	Plaur	NA	11291	0.7349716424942020	0.09453812	No
row_103	Gch1	NA	11432	0.7249846458435060	0.08812045	No
row_104	Klf6	NA	11615	0.7114217877388	0.078798436	No
row_105	Ptpre	NA	11643	0.7094613313674930	0.07997444	No
row_106	Ahr	NA	11686	0.7066797614097600	0.08012193	No
row_107	Adm	NA	11732	0.7040778398513790	0.080055036	No
row_108	Atp2c1	NA	11836	0.696780264377594	0.07602582	No
row_109	Il18	NA	11938	0.6891886591911320	0.072100006	No
row_110	Csf3r	NA	11942	0.6890894770622250	0.07481648	No
row_111	Itgb8	NA	11992	0.6853498220443730	0.074399106	No
row_112	Abi1	NA	12052	0.6817529201507570	0.073288664	No
row_113	Mefv	NA	12155	0.6741054058074950	0.06923115	No
row_114	Eif2ak2	NA	12341	0.6594001650810240	0.059485357	No
row_115	Slc31a1	NA	12397	0.6549477577209470	0.05853246	No
row_116	Nod2	NA	12440	0.6516565084457400	0.0584468	No
row_117	Ifitm1	NA	12526	0.6440563797950750	0.05541427	No
row_118	Slc31a2	NA	12532	0.6434206962585450	0.057801675	No
row_119	Acvr2a	NA	12540	0.643205463886261	0.060052603	No
row_120	Il12b	NA	12563	0.6407590508461000	0.06127642	No
row_121	Atp2a2	NA	12731	0.6270534992218020	0.052613657	No
row_122	Dcbld2	NA	12829	0.6172511577606200	0.048654154	No
row_123	Tnfrsf1b	NA	12856	0.6148384213447570	0.049497012	No
row_124	Fzd5	NA	12919	0.6095911860466000	0.04787745	No
row_125	Ptger2	NA	12982	0.6031436324119570	0.046230573	No
row_126	Tlr1	NA	12987	0.6027506589889530	0.04851343	No
row_127	Slc7a1	NA	13056	0.59597247838974	0.04642947	No
row_128	Gna15	NA	13123	0.5902875065803530	0.044456985	No
row_129	Kif1b	NA	13194	0.5828428864479070	0.042181823	No
row_130	Kcnj2	NA	13222	0.5806419849395750	0.042811997	No
row_131	Atp2b1	NA	13236	0.5795779824256900	0.044386625	No
row_132	Cybb	NA	13345	0.5672761797904970	0.039469756	No
row_133	Cd14	NA	13388	0.5625154972076420	0.039006382	No
row_134	Il18rap	NA	13450	0.5566343665122990	0.037230216	No
row_135	Msr1	NA	13468	0.554738461971283	0.03842846	No
row_136	Lcp2	NA	13485	0.5537927746772770	0.03969048	No
row_137	Tnfsf10	NA	13490	0.5534729361534120	0.04176454	No
row_138	Gnai3	NA	13562	0.5459622740745540	0.039265323	No
row_139	Sell	NA	13615	0.5409061908721920	0.038032558	No
row_140	Cxcr6	NA	13630	0.5390596389770510	0.039367717	No
row_141	Il18r1	NA	13655	0.5359023809432980	0.040011667	No
row_142	Cmklr1	NA	13721	0.5265215039253240	0.037836775	No
row_143	Cxcl10	NA	13797	0.516516387462616	0.03494166	No
row_144	Nampt	NA	13822	0.5126628279685970	0.035487138	No
row_145	Aqp9	NA	13961	0.4927716851234440	0.028221095	No
row_146	C3ar1	NA	14038	0.4820811152458190	0.025112286	No
row_147	Cd69	NA	14081	0.4743240475654600	0.024275225	No
row_148	Mxd1	NA	14141	0.4643620252609250	0.022243645	No
row_149	Raf1	NA	14249	0.4400912821292880	0.01685565	No
row_150	Sema4d	NA	14259	0.43763813376426700	0.018099977	No
row_151	Tlr2	NA	14281	0.4335244596004490	0.018513478	No
row_152	Rasgrp1	NA	14333	0.4197941720485690	0.016835317	No
row_153	Met	NA	14363	0.4140065312385560	0.016623853	No
row_154	Slc28a2	NA	14501	0.37426573038101200	0.008923455	No
row_155	Fpr1	NA	14528	0.3668241798877720	0.008715418	No
row_156	Irak2	NA	14554	0.3566414415836330	0.008532018	No
row_157	Rnf144b	NA	14563	0.35358813405036900	0.009487988	No
row_158	Ptafr	NA	14584	0.34085434675216700	0.009576608	No
row_159	Csf1	NA	14586	0.34068048000335700	0.010952366	No
row_160	Il15	NA	14631	0.32558268308639500	0.009349489	No
row_161	Il7r	NA	14665	0.3139076232910160	0.008442753	No
row_162	Il1b	NA	14707	0.29360201954841600	0.006907715	No
row_163	Clec5a	NA	14755	0.2713261842727660	0.004871593	No
row_164	Cxcl11	NA	14760	0.26882970333099400	0.0057395534	No
row_165	Il1a	NA	14768	0.2652212679386140	0.006388876	No
row_166	Osm	NA	14822	0.2288685441017150	0.0037661542	No
row_167	Olr1	NA	14852	0.20249687135219600	0.002658477	No
row_168	Ccrl2	NA	14871	0.16778315603733100	0.0021493214	No
row_169	Nlrp3	NA	14883	0.15074418485164600	0.0020424474	No
row_170	Sgms2	NA	14889	0.14187945425510400	0.0023047086	No
